# Synthesis and evaluation of anticancer, antiphospholipases, antiproteases, and antimetabolic syndrome activities of some 3*H-*quinazolin-4-one derivatives

**DOI:** 10.1080/14756366.2019.1574780

**Published:** 2019-03-01

**Authors:** Nahed N. E. El-Sayed, Norah M. Almaneai, Abir Ben Bacha, Omar Al-Obeed, Rehan Ahmad, Maha Abdulla, Ahmed M. Alafeefy

**Affiliations:** a Department of Chemistry, College of Science, King Saud University, Riyadh, Saudi Arabia;; b National Organization for Drug Control and Research, Giza, Egypt;; c Biochemistry Department, College of Sciences, King Saud University, Riyadh, Saudi Arabia;; d Department of Surgery, College of Medicine, King Saud University, Riyadh, Saudi Arabia;; e Department of Chemistry, Kulliyyah of Science, International Islamic University Malaysia, Kuantan, Pahang Darul Makmur, Malaysia

**Keywords:** *3H*-quinazolin-4-one, colorectal cancer, phospholipases, proteases, metabolic syndrome

## Abstract

Some new 3*H*-quinazolin-4-one derivatives were synthesised and screened for anticancer, antiphospholipases, antiproteases, and antimetabolic syndrome activities. Compound **15d** was more potent in reducing the cell viabilities of HT-29 and SW620 cells lines to 38%, 36.7%, compared to 5-FU which demonstrated cell viabilities of 65.9 and 42.7% respectively. The IC_50_ values of **15d** were ∼20 µg/ml. Assessment of apoptotic activity revealed that **15d** decreased the cell viability by down regulating Bcl2 and BclxL. Moreover, compounds, **8j**, **8d**/**15a**/**15e**, **5b,** and **8f** displayed lowered IC_50_ values than oleanolic acid against proinflammatory isoforms of hGV, hG-X, NmPLA_2,_ and AmPLA_2_. In addition, **8d**, **8h**, **8j**, **15a**, **15b**, **15e**, and **15f** showed better anti-α-amylase than quercetin, whereas **8g**, **8h**, and **8i** showed higher anti-α-glucosidase activity than allopurinol. Thus, these compounds can be considered as potential antidiabetic agents. Finally, none of the compounds showed higher antiproteases or xanthine oxidase activities than the used reference drugs.

## Graphical abstract

**Figure:**
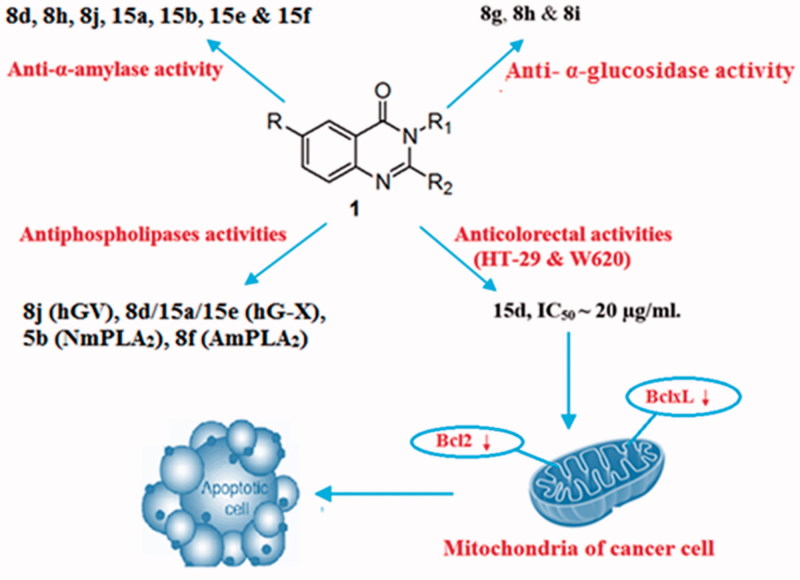

## Introduction

Colorectal cancer (CRC) is the third-most common cancer and the fourth-most common cause of cancer-related death worldwide^1^. One of the key features of cancer is deregulation of apoptosis of the altered cells^2^. Apoptosis is mediated by a complex network of BCL-2 family of proteins, comprising two types of proteins, namely anti-apoptotic proteins, which include Bcl2, BclxL, and Mcl-1, and pro-apoptotic proteins, which include Bax and Bak^3^. Hence, inhibiting the anti-apoptotic BCL-2 proteins can be established as a mechanism of anticancer defence for the treatment of CRC^4^.

Several *in vitro* and *in vivo* clinical studies have suggested a positive correlation between the activity of certain proteases, such as trypsin^5^, cathepsin B^6^, collagenases^7^, thrombin^8^, and neutrophil elastase^9^ and colorectal carcinogenesis, invasion, proliferation, and metastasis.

Furthermore, three phospholipases, namely (hsPLA_2_-IIA), group V, and group X^10^ are found to paly indirect pro-tumorigenic roles by promoting chronic inflammatory bowel diseases (IBD) that are common risk factors associated with CRC^11^.

In addition, recent studies have linked the incidence of CRC to certain metabolic syndrome components, especially elevated plasma glucose (type 2 diabetes mellitus)^12^. Several enzymes are reported to be involved in glucose homeostasis, among them α-amylase, α-glucosidase^13^, and xanthine oxidase^14^. Hence, targeting these enzymes has high potential in preventing CRC.

The 3*H*-quinazolin-4-one scaffold is known to be an important building block of various bioactive natural products^15^. In addition to this, many marketed drugs or experimental drug candidates incorporate this pharmacophore as part of the overall topography of the molecules^16^ such as ispinesib, nolatrexed, and raltitrexed (the anticancer agents); afloqualone with sedative and muscle relaxant effects, balaglitazone with antidiabetic activities, proquazone (as non-steroidal anti-inflammatory agent), and elinogrel (as reversible P2Y12 receptor antagonist) which is used for the treatment of acute coronary syndrome. Moreover, several new methodologies have been developed for the synthesis of 4-quinazolinone derivatives either using one pot multicomponent approaches^17^ or *via* multistep routes for investigating their pharmacological potential as dual EGFR/HER2 inhibitors^18^, phosphoinositide 3-kinase inhibitors^19^, and as antihypertensive^20^, antioxidant^21^, anticonvulsant^22^, and anti-inflammatory^23^ agents.

Based on these observations, and in continuation to our ongoing research programme aims to identify new bioactive molecules^24^ ^,^ ^25^ herein, we report the synthesis, characterisation, and cytotoxicity screening (including evaluation of apoptotic activity), for a new series of 3*H*-quinazolin-4-one derivatives against HT-29 and SW620 cell lines derived from CRC. Furthermore, the inhibitory potentials of the synthesised compounds against certain phospholipases and proteases implicated in cancer and inflammatory disorders, in addition to α-amylase, α-glucosidase, and xanthine oxidase, managing metabolic syndrome are investigated with the aim of eradicating the risk factors for CRC or combatting the disease.

## Materials and methods

### Chemistry

The reactions were monitored and the purity of the final products was checked using thin layer chromatography (TLC) on pre-coated silica gel sheets (60 F254, Merck, Kenilworth, NJ). The developed plates were examined by exposure to ultraviolet light using VL-6.LC (254, 365 nm, 50/60 Hz). All the melting points were determined in open capillary tubes with a Gallenkamp melting point apparatus (°C). The infra-red (IR) spectra were recorded on a Perkin Elmer Fourier transform infra-red (FTIR) spectrophotometer (Spectrum BX 1000) in wave numbers (cm^−1^) with potassium bromide (KBr) discs. Nuclear magnetic resonance (NMR) spectra were recorded on a Bruker NMR spectrometer: 1-Ascend 850 MHz for ^1^H and 213 MHz for ^13^C, or on 2-Avance 600 MHz for ^1^H and 150 MHz for ^13^C (Nuclear Magnetic Resonance Center, KAU, Jeddah, KSA), or on a Bruker Avance 500 spectrometer operating at 500 MHz for ^1^H and 125 MHz for ^13^C at 25 °C (Research Unit, College of Pharmacy, Prince Sattam Bin Abdulaziz University, AlKharj, KSA), or on an Eclipse 300 FT NMR spectrometer operating at 300 MHz spectrometer for ^1^H and at 75 MHz for ^13^C at 25 °C (KSU, Riyadh, KSA). The chemical shifts were expressed in (ppm) using tetramethylsilane as the internal standard; coupling constants (*J*) were expressed in Hz. Deuterochloroform (CDCl_3_) was used as solvent. The splitting patterns (multiplicities) in ^1^HNMR were designated as: singlet (s), broad singlet (br. s), doublet (d), double of doublet (dd), triplet (t), and multiplet (m). Mass spectra were recorded on Shimadzu Qp-2010 Plus mass spectrometer in ionisation mode: EI (Micro Analytical Center, Cairo University, Egypt). Elemental analyses were obtained from the Microanalytical Data Unit at Cairo University, Giza, Egypt.

Compounds **3a**,**b** ^26^, **3c** ^27^, **3d** ^28^, **6a** ^29^, **6b** ^30^, **13a**, **13b**, and **15a** ^31^ were reported previously.

### General procedure for the preparation of compounds 5a–c^*32*^ ^,^ ^*33*^

A mixture of the appropriate 2-methyl-benzo[*d*][1,3]oxazin-4-one derivative **3a-c** (0.003 mol) and 3,4,5-trimethoxyaniline **4** (0.003 mol, 0.55 g) was fused in oil bath at 200 °C for 2 h. After cooling down the temperature to 100 °C, 37% HCl (Conc) (3 ml) was added till all the material was dissolved completely. The reaction mixture was cooled down to room temperature and was made alkaline with diluted NaOH solution (25%). The crude precipitated solid in each case, was filtered, washed with water, dried, and recrystallised from ethanol to yield compounds **5a–c**.

### 2 -Methyl-3-(3,4,5-trimethoxy-phenyl)-3H-quinazolin-4-one (5a)

Black powder, yield (53%), mp 88–90 °C; ν_max_ (KBr)/cm^−1^ 3080 (CH-Ar), 2935 & 2837 (CH-aliphatic), 2368, 1682 (C = O), 1600 & 1503 (C = C), 1465, 1422, 1376, 1321, 1233, 1124, 1002, 936, 835, 771, 700, 653, 524; δ_H_ (850 MHz; CDCl_3_) 8.26 (1H, d, *J*= 7.6, CH^5^-quinazolin-4-one), 7.77–7.75 (1H, m, CH^6^-quinazolin-4-one), 7.66 (1H, dd, *J*= 3.4, 8.5, CH^8^-quinazolin-4-one), 7.47–7.45 (1H, m, CH^7^-quinazolin-4-one), 6.47 (2H, s, H^2^'^^ and H^6'^-trimethoxyphenyl), 3.90 (3H, s, OCH_3_), 3.84 (6H, s, 2 × OCH_3_), 2.32 (3H, s, CH_3_); δ_C_ (213 MHz; CDCl_3_) 162.36 (C = O), 154.41 (C = N), 154.22, 147.41, 138.46, 134.70, 133.29, 127.04, 126.79, 126.73, 120.69, 105.19 (6 × CH-Ar & 6 × C_q_-Ar), 60.96 (OCH_3_), 56.27 (2 × OCH_3_), 24.04 (CH_3_); MS (EI) *m/z* (%) [M^+^ + 2] 328.10 (2.50), [M^+^ + 1] 327.10 (16.69), [M^+^] 326.10 (77.52), 311.10 (46.20), 297.10 (3.85), 279.05 (2.13), 265.05 (1.90), 183.05 (4.87), 168.10 (7.32), 147.65 (8.78), 144.10 (14.54), 143.10 (100.00), 134.10 (4.69), 116.10 (6.24), 102.05 (4.96), 77.00 (6.38), 57.05 (3.38). Anal. calcd. for C_18_H_18_N_2_O_4_ (326.13): C, 66.25; H, 5.56; N, 8.58. Found: C, 66.23; H, 5.46; N, 8.66.

### 6 -Fluoro-2-methyl-3–(3,4,5-trimethoxy-phenyl)-3H-quinazolin-4-one (5b)

Black crystals, yield (55%), mp 193–195 °C; ν_max_ (KBr)/cm^−1^ 3079 (CH-Ar), 2931 & 2835 (CH-aliphatic), 2375, 2278, 1673 (C = O), 1601 & 1484 (C = C), 1425, 1374, 1339, 1313, 1235, 1183, 1125 (C–F), 1065, 1008, 966, 878, 843, 778, 752, 723, 674, 626, 558, 526, 458; δ_H_ (500 MHz; CDCl_3_) 7.84–7.82 (1H, m, CH^5^-quinazolin-4-one), 7.67–7.65 (1H, m, CH^7^-quinazolin-4-one), 7.49–7.45 (1H, m, CH^8^-quinazolin-4-one), 6.52 (2H, s, H^2^'^^ and H^6'^-trimethoxyphenyl), 3.91 (3H, s, OCH_3_), 3.86 (6H s, 2 × OCH_3_), 2.32 (3H, s, CH_3_); δ_C_ (125 MHz; CDCl_3_) 161.62 (C = O), 159.65 (C_q_-F), 154.20 (C = N), 153.65, 144.06, 138.48, 133.03, 129.23, 123.18, 122.99, 121.87, 105.12 (5 × CH-Ar & 6 × C_q_-Ar), 60.89 (OCH_3_), 56.24 (2 × OCH_3_), 23.87 (CH_3_); MS (EI) *m/z* (%) [M^+^ + 1] 345.10 (21.69), [M^+^] 344.10 (100) 330.10 (11.87), 329.10 (57.89), 313.10 (3.99), 297.05 (2.71), 161.05 (86.15), 162.05 (11.84), 134.10 (7.80), 107.05 (4.64), 94.05 (7.76), 77.00 (1.91), 57.00 (3.40). Anal. calcd. for C_18_H_17_FN_2_O_4_ (344.12): C, 62.79; H, 4.98; N, 8.14. Found: C, 62.68; H, 4.63; N, 8.02.

### 6 -Chloro-2-methyl-3–(3,4,5-trimethoxy-phenyl)-3H-quinazolin-4-one (5c)

Black powder, yield (60%), mp 188–190 °C; ν_max_ (KBr)/cm^−1^ 3075 (CH-Ar), 2934 & 2839 (CH-aliphatic), 2369, 1680 (C = O), 1598 & 1504 (C = C), 1467, 1423, 1375, 1312, 1236, 1126, 998, 948, 892, 834 (C–Cl), 771, 738, 692, 613, 524, 452; δ_H_ (500 MHz; CDCl_3_) 8.18 (1H, d, *J*= 2.4, CH^5^-quinazolin-4-one), 7.69 (1H, dd, *J*= 8.7, 2.4, CH^7^-quinazolin-4-one), 7.60 (1H, d, *J*= 8.7, CH^8^-quinazolin-4-one), 6.51 (2H, s, H^2'^ and H^6'^-trimethoxyphenyl), 3.92 (3H, s, OCH_3_), 3.88 (6H, s, 2 × OCH_3_), 2.32 (3H, s, CH_3_); δ_C_ (125 MHz; CDCl_3_) 161.28 (C = O), 154.75 (C = N), 154.25, 145.91, 138.56, 135.02, 132.95, 132.36, 128.52, 126.23, 121.73, 105.09 (5 × CH-Ar & 7 × C_q_-Ar), 60.93 (OCH_3_), 56.28 (2 × OCH_3_), 24.02 (CH_3_); MS (EI) *m/z* (%) [M^+^, Cl^37^] 362.10 (34.88), [M^+^, Cl^35^] 360.10 (100.00), 347.05 (22.20), 345.10 (60.80), 331.05 (4.80), 329.05 (4.53), 315.05 (1.90), 313.05 (3.09), 301.05 (1.04), 299.10 (1.56), 179.00 (27.46), 177.00 (84.58), 152.05 (3.81), 150.05 (6.54), 136.05 (5.31), 123.05 (3.29), 110.00 (6.17), 75.00 (6.71), 57.10 (3.05). Anal. calcd. for C_18_H_17_ClN_2_O_4_ (360.09): C, 59.92; H, 4.75; N, 7.76. Found: C, 59.88; H, 4.62; N, 7.59.

### Synthesis of Schiff’s base derivatives (8a–j)^*34*^

A mixture of 3-amino-2-methyl-3*H*-quinazolin-4-one derivatives **6a**,**b** (0.002 mol) and the appropriate aromatic aldehyde **7a–e**, namely, 2-naphthaldehyde, 4-(pyridin-2-yl) benzaldehyde, 3-methoxy-2-nitrobenzaldehyde, 4-formylbenzonitrile, and 1,1′-biphenyl]-4-carbaldehyde (0.002 mol) in absolute ethanol (20 ml) containing (3 ml) glacial acetic acid was heated under reflux for 10 h. The excess solvent was removed under reduced pressure. The obtained solid in each case was washed with water, air dried, and recrystallised from the appropriate solvent to obtain the corresponding Schiff's base.

### (E)-2-Methyl-3-[(naphthalen-2-ylmethylene)-amino]-3H-quinazolin-4-one (8a)

White powder (ethanol), yield (74%), mp 163–165 °C; ν_max_ (KBr)/cm^−1^; 3062 (CH-Ar), 2929 (CH-alipahtic), 2371, 2340, 1667 (C = O), 1596 & 1468 (C = C), 1426, 1377, 1346, 1320, 1268, 1227, 1156, 1121, 1031, 934, 898, 866, 831, 778, 755, 693, 660, 621, 477; δ_H_ (600 MHz; CDCl_3_) 9.18 (1H, s, CH = N), 8.30 (1H, d, *J*= 8.4, CH-Ar), 8.16–8.14 (2H, m, 2 × CH-Ar), 7.93–7.92 (2H, m, 2 × CH-Ar), 7.89 (1H, d, *J*= 7.8, CH-Ar), 7.75 (1H, d, *J*= 7.2, CH-Ar), 7.67 (1H, d, *J*= 8.4, CH-Ar), 7.59 (1H, t, *J*= 7.2, CH-Ar), 7.55 (1H, t, *J*= 7.8, CH-Ar), 7.47 (1H, t, *J*= 7.8, CH-Ar), 2.70 (3H, s, CH_3_); δ_C_ (150 MHz; CDCl_3_) 166.32 (C = O), 158.84 (HC = N), 154.21 (C_q_=N), 146.49, 135.41, 134.37, 132.93, 132.42, 130.46, 128.97, 128.95, 128.20, 128.02, 127.30, 126.95, 126.94, 126.46, 122.88, 121.58 (11 × CH-Ar & 5 × C_q_-Ar), 23.00 (CH_3_); MS (EI) *m/z* (%) [M^+^ + 1] 314.10 (0.44), [M^+^] 313.10 (1.41), 161.05 (10.24), 160.10 (100.00), 153.10 (14.99), 139.10 (4.10), 127.10 (8.79), 118.10 (11.47), 90.05 (11.47), 76.00 (13.65), 63.00 (2.80), 50.00 (3.12). Anal. calcd. for C_20_H_15_N_3_O (313.12): C, 76.66; H, 4.82; N, 13.41. Found: C, 76.62; H, 4.77; N, 13.29.

### (E)-6-Bromo-2-methyl-3-[(naphthalen-2-ylmethylene)-amino]-3H-quinazolin-4-one (8b)

White powder (ethanol), yield (65%), mp 200 °C; ν_max_ (KBr)/cm^−1^ 3056 (CH-Ar), 2926 (CH-aliphatic), 1932, 1675 (C = O), 1602 & 1465 (C = C), 1371, 1347, 1313, 1272, 1235, 1208, 1180, 1153, 1122, 1072, 1037, 954, 890, 860, 831, 812, 777, 740, 700, 672 (C-Br), 629, 569, 539, 514, 473; δ_H_ (500 MHz; CDCl_3_) 9.15 (1H, s, CH = N), 8.39 (1H, s, CH-Ar), 8.15 (1H, s, CH-Ar), 8.12 (1H, d, *J*= 8.5, CH-Ar), 7.92 (2H, d, *J*= 8.2, 2 × CH-Ar), 7.88 (1H, d, *J*= 7.9, CH-Ar), 7.79 (1H, dd, *J*= 8.5, 1.8, CH-Ar), 7.61–7.53 (3H, m, 3 × CH-Ar), 2.68 (3H, s, CH_3_); δ_C_ (125 MHz; CDCl_3_) 166.60 (C = O), 157.65 (HC = N), 154.66 (C_q_=N), 145.31, 137.46, 135.46, 132.90, 132.56, 130.26, 129.69, 128.98, 128.80, 128.28, 128.01, 126.98, 122.98, 122.81, 119.86 (10 × CH-Ar & 6 × C_q_-Ar), 22.98 (CH_3_); MS (EI) *m/z* (%) [M^+^, ^81^Br] 393.05 (2.94), [M^+^, ^79^Br] 391.00 (2.85), 239.95 (100.00), 237.95 (98.69), 197.90 (6.54), 195.95 (4.69), 155.95 (5.37), 153.05 (22.90), 127.10 (21.27), 100.05 (3.19), 75.00 (18.97), 63.00 (3.47), 57.05 (1.26). Anal. calcd. for C_20_H_14_BrN_3_O (391.03): C, 61.24; H, 3.60; N, 10.71. Found: C, 61.08; H, 3.39; N, 10.65.

### (E)-2-Methyl-3-[(4-pyridin-2-yl-benzylidene)-amino]-3H-quinazolin-4-one (8c)

Beige powder (benzene), yield (85%), mp 188–190 °C; ν_max_ (KBr)/cm^−1^ 3042 (CH-Ar), 2940 (CH-aliphatic), 1675 (C = O), 1600 & 1468 (C = C), 1436, 1378, 1327, 1230, 1181, 1154, 1108, 987, 939, 875, 847, 772, 718, 688, 663, 626, 589, 479; δ_H_ (500 MHz; CDCl_3_) 9.12 (1H, s, CH = N), 8.74 (1H, d, *J*= 4.7, CH-Ar), 8.30 (1H, d, *J*= 7.9, CH-Ar), 8.15 (2H, d, *J*= 8.2, 2 × CH-Ar), 8.00 (2H, d, *J*= 8.2, 2 × CH-Ar), 7.80–7.73 (3H, m, 3 × CH-Ar), 7.68 (1H, d, *J*= 8.1, CH-Ar), 7.48 (1H, t, *J*= 7.5, CH-Ar), 7.36–7.28 (1H, m, CH-Ar), 2.69 (3H, s, CH_3_); δ_C_ (125 MHz; CDCl_3_) 165.64 (C = O), 158.80 (HC = N), 156.12 (HC = N), 154.18 (C_q_=N), 149.93, 146.46, 143.05, 136.92, 134.34, 133.19, 129.24, 127.35, 127.27, 126.93 126.43, 122.87, 121.56, 120.88 (11 × CH-Ar & 5 × C_q_-Ar), 22.94 (CH_3_); MS (EI) *m/z* (%) [M^+^ + 1] 341.10 (0.69), [M^+^] 340.10 (1.39), 181.05 (4.88), 161.05 (10.51), 160.10 (100.00), 145.10 (2.83), 132.10 (3.91), 118.10 (12.08), 90.00 (5.53), 76.00 (16.01), 50.00 (3.71). Anal. calcd. for C_21_H_16_N_4_O (340.13): C, 74.10; H, 4.74; N, 16.46. Found: C, 73.92; H, 4.63; N, 16.28.

### (E)-6-Bromo-2-methyl-3-[(4-pyridin-2-yl-benzylidene)-amino]-3H-quinazolin-4-one (8d)

Beige powder (benzene), yield (77%), mp 209–210 °C; ν_max_ (KBr)/cm^−1^ 3149 & 3060 (CH-Ar), 2952 (CH-aliphatic), 1670 (C = O), 1604 & 1465 (C = C), 1375, 1319, 1278, 1240, 1208, 1158, 1124, 1961, 1035, 987, 876, 833, 775, 727, 674 (C-Br), 645, 564; δ_H_ (850 MHz; CDCl_3_) 9.10 (1H, s, CH = N), 8.77 (1H, d, *J*= 4.3, CH-Ar), 8.41 (1H, d, *J*= 2.6, CH-Ar), 8.17 (2H, d, *J*= 7.7, 2 × CH-Ar), 8.01 (2H, d, *J*= 7.7, 2 × CH-Ar), 7.85–7.79 (3H, m, 3 × CH-Ar), 7.55 (1H, d, *J*= 8.5, CH-Ar), 7.36–7.32 (1H, m, CH-Ar), 2.68 (3H, s, CH_3_); δ_C_ (213 MHz; CDCl_3_) 165.98 (C = O), 157.67 (HC = N), 155.79 (HC = N), 154.69 (C_q_=N), 149.51, 145.26, 137.56, 133.23, 129.74, 129.40, 128.79, 127.54, 123.08, 122.95, 121.23, 119.94 (10 × CH-Ar & 6 × C_q_-Ar), 22.95 (CH_3_); MS (EI) *m/z* (%) [M^+^ + 1] 419.05 (1.37), [M^+^] 418.00 (2.03), 240.00 (11.07), 239.95 (100.00%), 237.95 (98.10), 199.90 (2.69), 197.90 (6.83), 181.05 (6.55), 179.05 (4.60), 154.05 (13.02), 152.05 (1.88), 139.10 (3.04), 127.10 (6.15), 116.10 (3.42), 90.05 (4.15), 78.05 (3.66), 75.00 (17.16). Anal. calcd. for C_21_H_15_^79^BrN_4_O (418.04): C, 60.16; H, 3.61; N, 13.36. Found: C, 60.03; H, 3.43; N, 13.11.

### (E)-3-[(3-Methoxy-2-nitro-benzylidene)-amino]-2-methyl-3H-quinazolin-4-one (8e)

Beige powder (ethanol), yield (55%), mp 178–180 °C; ν_max_ (KBr)/cm^−1^ 3017 (CH-Ar), 2975 & 2935 (CH-aliphatic), 1680 (C = O), 1615 & 1571 (C = C), 1537, 1464, 1428, 1376, 1283, 1227, 1156, 1107, 1055, 1029, 964, 933, 875, 850, 772, 743, 691, 651, 589, 471, 419; δ_H_ (850 MHz; CDCl_3_) 9.41 (1H, s, CH = N), 8.26 (1H, d, *J*= 7.7, CH-Ar), 7.75 (1H, t, *J*= 7.7, CH-Ar), 7.67 (1H, d, *J*= 7.7, CH-Ar), 7.56 (1H, t, *J*= 7.7, CH-Ar), 7.49–7.46 (2H, m, 2 × CH-Ar), 7.23 (1H, d, *J*= 8.5, CH-Ar), 3.96 (3H, s, OCH_3_), 2.6 (3H, s, CH_3_); δ_C_ (213 MHz; CDCl_3_) 159.22 (C = O), 158.52 (HC = N), 154.87 (C_q_=N), 151.44, 139.74, 134.76, 131.30, 127.39, 126.77, 126.14, 122.03, 121.38, 115.92 (7 × CH-Ar & 5 × C_q_-Ar), 56.78 (OCH_3_), 22.65 (CH_3_); MS (EI) *m/z* (%) [M^+^] 338.10 (4.91), 321.10 (8.62), 290.10 (8.60), 261.10 (2.25), 186.05 (2.04), 160.10 (100.00), 143.10 (3.47), 132.10 (4.81), 117.10 (18.04), 104.05 (3.96), 76.00 (26.95), 63.00 (4.85), 50.00 (6.06). Anal. calcd. for C_17_H_14_N_4_O_4_ (338.10): C, 60.35; H, 4.17; N, 16.56. Found: C, 60.03; H, 3.94; N, 16.37.

### (E)-6-Bromo-3-[(3-methoxy-2-nitro-benzylidene)-amino]-2-methyl-3H-quinazolin-4-one (8f)

Beige powder (ethanol), yield (66%), mp 234–235 °C; ν_max_ (KBr)/cm^−1^ 3070 & 3012 (CH-Ar), 2977 & 2926 (CH-aliphatic), 1677 (C = O), 1601 & 1533 (C = C), 1464, 1436, 1373, 1303, 1207, 1153, 1104, 1063, 973, 937, 897, 836, 779, 739, 670 (C-Br), 639, 589; δ_H_ (850 MHz; CDCl_3_) 9.35 (1H, s, CH = N), 8.38 (1H, d, *J*= 2.6, CH-Ar), 7.82 (1H, dd, *J*= 8.5, 2.6, CH-Ar), 7.58–7.56 (2H, m, 2 × CH-Ar), 7.48 (1H, d, *J*= 8.5, CH-Ar), 7.25 (1H, d, *J*= 8.5, CH-Ar), 3.97 (3H, s, OCH_3_), 2.63 (3H, s, CH_3_); δ_C_ (213 MHz; CDCl_3_) 159.28 (C = O), 157.93 (HC = N), 155.38 (C_q_=N), 151.47, 144.56, 139.68, 137.94, 131.36, 129.85, 128.57, 125.86, 122.76, 122.14, 120.31, 116.16 (6 × CH-Ar & 6 × C_q_-Ar), 56.80 (OCH_3_), 22.63 (CH_3_); MS (EI) *m/z* (%) [M^+^, ^81^Br] 418.00 (4.65), [M^+^, ^79^Br] 416.00 (4.58), 401.00 (6.90), 399.00 (6.63), 370.00 (5.89), 368.00 (5.13), 341.00 (1.54), 334.00 (1.46), 265.95 (2.41), 263.95 (2.42), 239.95 (97.78), 237.95 (100.00), 197.90 (8.61), 195.95 (6.33), 169.95 (3.24), 167.95 (2.54), 155.95 (7.13), 153.95 (7.22), 133.10 (1.53), 131.10 (3.71), 117.10 (7.38), 116.10 (5.63), 101.10 (4.34), 89.00 (4.73), 76.00 (9.56), 59.05 (13.18), 57.05 (4.20). Anal. calcd. for C_17_H_13_^79^BrN_4_O_4_ (416.01): C, 48.94; H, 3.14; N, 19.15. Found: C, 48.83; H, 2.97; N, 18.93.

### (E)-4-[(2-Methyl-4-oxo-4H-quinazolin-3-ylimino)-methyl]-benzonitrile (8g)

White powder (acetone), yield (55%), mp 222–224 °C; ν_max_ (KBr)/cm^−1^ 3043 (CH-Ar), 2960 (CH-aliphatic), 2226 (CN), 1674 (C = O), 1599 & 1467 (C = C), 1406, 1376, 1320, 1235, 1145, 1111, 1034, 944, 868, 836, 766, 687, 660, 555; δ_H_ (500 MHz; CDCl_3_) 9.37 (1H, s, CH = N), 8.28 (1H, d, *J*= 8.0, CH^5^-quinazolin-4-one), 8.00 (2H, d, *J*= 8.2, 2 × CH-benzonitrile ring), 7.80 (2H, d, *J*= 8.2, 2 × CH-benzonitrile ring), 7.77 (1H, app. t, *J*= 7.1, CH^7^-quinazolin-4-one), 7.68 (1H, d, *J*= 8.0, CH^8^-quinazolin-4-one), 7.49 (1H, app. t, *J*= 7.6, CH^6^-quinazolin-4-one), 2.73 (3H, s, CH_3_); δ_C_ (125 MHz; CDCl_3_) 162.10 (C = O), 159.10 (HC = N), 154.14 (C_q_=N), 146.23, 137.25, 134.71, 132.69, 128.98, 127.34, 127.07, 126.75, 121.48, 118.16, 115.36 (8 × CH-Ar, 4 × C_q_-Ar & C≡N), 23.02 (CH_3_); (EI) *m/z* (%) [M^+^ + 2] 290.10 (3.21), [M^+^ + 1] 289.10 (1.58), [M^+^] 288.10 (5.65), 273.10 (3.00), 272.10 (6.19), 260.10 (1.60), 186.05 (3.69), 161.10 (10.65), 160.10 (100.00), 145.10 (4.68), 143.10 (3.86), 131.10 (14.47), 130.10 (17.06), 119.10 (11.52), 117.10 (19.54), 102.10 (18.65), 101.10 (5.04), 92.05 (5.14), 90.05 (11.61), 77.00 (20.32), 76.00 (37.53), 60.00 (4.25), 59.05 (19.07), 51.00 (6.85), 50.00 (14.71). Anal. calcd. for C_17_H_12_N_4_O (288.10): C, 70.82; H, 4.20; N, 19.43. Found: C, 70.69; H, 4.13; N, 19.27.

### (E)-4-[(6-Bromo-2-methyl-4-oxo-4H-quinazolin-3-ylimino)-methyl]-benzonitrile (8h)

White powder (acetone), yield (75%), mp 228–230 °C; ν_max_ (KBr)/cm^−1^ 3059 (CH-Ar), 2927 (CH-aliphatic), 2230 (CN), 1680 (C = O), 1602 & 1467 (C = C), 1420, 1376, 1328, 1277, 1242, 1211, 1156, 1123, 1071, 981, 899, 876, 831, 778, 723, 675 (C-Br), 644, 555, 433; δ_H_ (500 MHz; CDCl_3_) 9.32 (1H, s, CH = N), 8.40 (1H, s, CH^5^-quinazolin-4-one ring), 8.00 (2H, d, *J*= 7.7, 2 × CH-benzonitrile ring), 7.84 (1H, d, *J*= 8.5, CH^7^-quinazolin-4-one ring), 7.81 (2H, d, *J*= 7.7, 2 × CH-benzonitrile ring), 7.55 (1H, d, *J*= 8.5, CH^8^-quinazolin-4-one ring), 2.64 (3H, s, CH_3_); δ_C_ (125 MHz; CDCl_3_) 162.65 (C = O), 157.83 (HC = N), 154.53 (C_q_=N), 145.027, 137.87, 136.93, 132.67, 129.78, 129.01, 128.9, 122.81, 120.14, 118.04, 115.49 (7 × CH-Ar, 5 × C_q_-Ar & C≡N), 22.98 (CH_3_); MS (EI) *m/z* (%) [M^+^, ^81^Br] 368.01 (2.60), [M^+^, ^79^Br] 366.01 (2.56), 354.30 (3.01), 313.30 (12.92), 297.30 (5.50), 239.20 (12.54), 227.10 (2.29), 213.15 (2.33), 185.10 (2.94), 171.10 (2.88), 143.15 (3.48), 129.10 (12.26), 111.15 (14.46), 97.15 (21.74), 83.05 (26.49), 71.05 (43.57), 69.05 (42.50), 59.05 (56.18), 57.05 (100.00). Anal. calcd. for C_17_H_11_^79^BrN_4_O (366.01): C55.61; H, 3.02; N, 15.26. Found: C, 55.49; H, 2.88; N, 15.19.

### (E)-3-[(Biphenyl-4-ylmethylene)-amino]-6-bromo-2-methyl-3H-quinazolin-4-one (8j)

White powder (ethanol), yield (97%), mp 208–210 °C; ν_max_ (KBr)/cm^−1^ 3058 (CH-Ar), 2920 (CH-aliphatic), 1676 (C = O), 1604 & 1467 (C = C), 1373, 1319, 1276, 1236, 1206, 1180, 1154, 1074, 1007, 907, 875, 831, 757, 722, 689 (C-Br), 644, 555, 467; δ_H_ (500 MHz; CDCl_3_) 9.03 (1H, s, CH = N), 8.41 (1H, d, *J*= 2.3, CH-Ar), 7.96 (2H, d, *J*= 8.3, 2 × CH-Ar), 7.82 (1H, dd, *J*= 8.7, 2.3, CH-Ar), 7.73 (2H, d, *J*= 8.3, 2 × CH-Ar), 7.66 (2H, d, *J*= 7.3, 2 × CH-Ar), 7.54 (1H, d, *J*= 8.7, CH-Ar), 7.49 (2H, t, *J*= 7.3, 2 × CH-Ar), 7.41 (1H, t, *J*= 7.3 CH-Ar), 2.63 (3H, s, CH_3_); δ_C_ (125 MHz; CDCl_3_) 166.41 (C = O), 157.64 (HC = N), 154.61 (C_q_=N), 145.45, 145.34, 139.92, 137.49, 131.44, 129.71, 129.43, 129.02, 128.81, 128.25, 127.66, 127.23, 122.97, 119.87 (12 × CH-Ar & 6 × C_q_-Ar), 22.94 (CH_3_); MS (EI) *m/z* (%) [M^+^, ^81^Br] 419.05 (1.70), [M^+^, ^79^Br] 417.05 (1.67), 239.95 (100.00), 237.95 (98.77), 197.90 (6.94), 195.95 (4.46), 179.05 (24.10), 165.05 (5.04), 152.10 (13.04), 131.10 (2.31), 116.10 (3.58), 116.10 (3.58), 90.05 (3.62), 75.00 (15.91), 63.00 (3.20), 57.05 (2.43). Anal. calcd. for C_22_H_16_^79^BrN_3_O (417.05): C, 63.17; H, 3.86; N, 10.05. Found: C, 62.97; H, 3.63; N, 9.89.

### Synthesis of (2-methyl-4-oxo-4H-quinazolin-3-yl)-carbamic acid isobutyl ester (10)

A mixture of 3-amino-2-methyl-3*H*-quinazolin-4-one **6a** (0.0028 mol, 0.5 g) and isobutyl chloroformate **9** (13.6 equiv., 0.038 mol, 5.2 g, 4.94 ml) was refluxed for 10 h^35^. The excess reagent was evaporated under reduced pressure and the remaining residue was treated with diethyl ether. The precipitated solid was filtered, washed with water, and purified by recrystallisation from benzene to obtain the *title compound* as a beige powder with 50% yield, mp 168–170 °C; *v*_max_ (KBr)/cm^−1^ 3223 (NH), 3080 (CH-Ar), 2963 & 2875 (CH-aliphatic), 1755 (C = O), 1694 (C = O), 1651 (C = N), 1606 & 1508 (C = C), 1468, 1380, 1232, 1108, 1046, 988, 942, 872, 772, 719, 695, 633, 520, 462; δ_H_ (850 MHz; CDCl_3_) 8.19 (1H, d, *J*= 7.7, CH-Ar), 7.75 (1H, app. t, *J*= 7.7, CH-Ar), 7.64 (1H, d, *J*= 8.5, CH-Ar), 7.62 (1H, br. s, NH), 7.44 (1H, t, *J*= 7.7, CH-Ar), 4.00 (2H, apparent s, O– CH_2_– CH–(CH_3_)_2_), 2.60 (3H, s, CH_3_), 1.96–1.93 (1H, m, O-CH_2_-CH-(CH_3_)_2_), 0.94 (6H, d, *J*= 6.0, O– CH_2_–CH–(CH_3_)_2_); δ_C_ (125 MHz; CDCl_3_) 160.78 (C = O), 156.28 (C = O), 156.15 (C = N), 146.83, 134.89, 126.94, 126.85, 126.58, 120.60 (4 × CH-Ar, 2 × C_q_-Ar), 72.71 (OCH_2_), 27.78 (CH), 21.52 (CH_3_), 18.63 (2 × CH_3_); MS (EI) *m/z* (%) [M^+^ + 1] 276.10 (21.00), [M^+^] 275.10 (100.00), 260.10 (1.46), 220.05 (4.41), 202.00 (11.98), 175.05 (95.02), 160.05 (9.43), 146.10 (85.56), 131.10 (4.53), 131.10 (4.53), 117.10 (27.22), 90.00 (23.15), 76.00 (30.46), 63.00 (8.24), 57.05 (86.01). Anal. calcd. for C_14_H_17_N_3_O_3_ (275.13): C, 61.08; H, 6.22; N, 15.26. Found: C, 61.00; H, 6.01; N, 15.17.

### Synthesis of (E)-N,N-dimethyl-N′-(2-methyl-4-oxo-4H-quinazolin-3-yl)-formamidine (12)

A mixture of 3-amino-2-methyl-3*H*-quinazolin-4-one **6a** (0.0028 mol, 0.5 g) and *N,N*-dimethyl formamide dimethyl acetal **11** (5.39 equiv. 0.0151 mol, 1.79 g, 2 ml) in ethanol (15 ml) was refluxed for 2 h^36^. The excess solvent was removed under reduced pressure. The remaining solid was washed with *n*-hexane and recrystallised from ethanol to obtain the *title compound* as a yellow powder with 50% yield, mp 100–102 °C; ν_max_ (KBr)/cm^−1^ 3010 (CH-Ar), 2924 & 2807 (CH-aliphatic), 2145, 1660 (C = O), 1621 (C = N), 1609 & 1589 (C = C), 1475, 1418, 1366, 1323, 1237, 1174, 1120, 1069, 944, 874, 783, 701, 660, 626, 551, 509; δ_H_ (500 MHz; CDCl_3_) 8.23 (1H, dd, *J*= 7.7, 1.7, CH-Ar), 7.74 (1H, s, HC = N), 7.69–7.67 (1H, m, CH-Ar), 7.62 (1H, dd, *J*= 8.5, 2.6, CH-Ar), 7.41–7.39 (1H, m, CH-Ar), 3.08 (3H, s, N-CH_3_), 3.04 (3H, s, N-CH_3_), 2.52 (3H, s, CH_3_); δ_C_ (125 MHz; CDCl_3_) 162.22 (C = O), 160.08 (HC = N), 155.08 (C_q_=N), 146.39, 133.39, 126.41, 125.61, 121.04 (4 × CH-Ar & 2 × C_q_-Ar), 40.88, 34.53 (N(CH_3_)_2_), 22.56 (CH_3_); MS (EI) *m/z* (%) [M^+^ + 1] 231.10 (5.52), [M^+^] 230.10 (34.52), 215.05 (1.48), 186.00 (10.30), 161.10 (11.53), 160.10 (100.00), 145.10 (5.33), 132.10 (7.42), 118.10 (21.02), 104.10 (1.60), 90.00 (9.13), 76.00 (16.94), 63.00 (5.01), 50.00 (6.27). Anal. calcd. for C_12_H_14_N_4_O (230.12): C, 62.59; H, 6.13; N, 24.33. Found: C, 62.47; H, 5.98.; N, 24.18.

### Synthesis of 2-(4-chloro-benzylsulfanyl)-6-methyl-3-phenyl-3H-quinazolin-4-one (15a^*31*^, 15b) and 2-(4-oxo-3-phenyl-3,4-dihydro-quinazolin-2-ylsulfanyl)-acetamide derivatives (15c–f)

A mixture of the appropriate derivative of 2-mercapto-3-phenyl-3*H*-quinazolin-4-one (**13a'**,**b')** (0.0018 mol) and 4-chlorobenzyl chloride **14a** or chloroacetamide derivatives **14b**,**c** ^37^ ^,^ ^38^ (1 equiv., 0.0018 mol) and potassium carbonate (2.5 equiv., 0.0045 mol, 0.62 g) in dry acetone (20 ml) was refluxed for 6 h^39^. The hot reaction mixture was filtered and the filtrate was concentrated under reduced pressure. The resulting solid in each case was washed with water, air dried, and recrystallised from ethanol to obtain compounds **15a**–**f**.

### 2 -(4-Chloro-benzylsulfanyl)-6-methyl-3-phenyl-3H-quinazolin-4-one (15b)

White powder, yield (96%), mp 164–165 °C; ν_max_ (KBr)/cm^−1^ 3058 (CH-Ar), 2915 (CH-aliphatic), 1689 (C = O), 1617 & 1575 (C = C), 1548, 1487, 1401, 1399, 1335, 1299, 1259, 1204, 1128, 1089, 1014, 963, 824 (C–Cl), 742, 692, 641, 539, 506; δ_H_ (500 MHz; CDCl_3_) 8.01 (1H, s, CH^5^-quinazolin-4-one), 7.54–7.48 (5H, m, 5 × CH-Ar), 7.29–7.18 (6H, m, 6 × CH-Ar), 4.32 (2H, s, CH_2_), 2.44 (3H, s, CH_3_); δ_C_ (125 MHz; CDCl_3_) 161.83 (C = O), 155.60 (C = N), 145.82, 136.17, 136.12, 135.90, 135.38, 133.28, 130.77, 130.00, 129.72, 129.26, 128.68, 126.76, 126.05, 119.72 (12 × CH-Ar & 6 × C_q_-Ar), 36.22 (CH_2_), 21.33 (CH_3_); MS (EI) *m/z* (%) [M^+^, ^37^Cl] 394.05 (27.71), [M^+^ + 1, ^35^Cl] 393.10 (19.45), [M^+^, ^35^Cl] 392.10 (69.22), 375.05 (1.96), 361.10 (35.50), 359.10 (100.00), 324.10 (11.09), 301.00 (12.62), 283.05 (37.48), 267.05 (81.50), 249.10 (51.70), 235.05 (33.05), 201.00 (89.80), 193.05 (3.31), 180.05 (4.87), 166.05 (14.31), 150.05 (8.84), 133.10 (77.88), 104.10 (28.46), 89.00 (25.12), 77.00 (63.94), 63.00 (10.37), 51.00 (11.84). Anal. calcd. for C_22_H_17_^35^ClN_2_OS (392.08): C, 67.25; H, 4.36; N, 7.13. Found: C, 67.20; H, 4.32; N, 7.06.

### N-(2,6-Dimethyl-phenyl)-2-(4-oxo-3-phenyl-3,4-dihydro-quinazolin-2-ylsulfanyl)-acetamide (15c)

White crystals, yield (55%), mp 218–220 °C; ν_max_ (KBr)/cm^−1^ 3331 (NH), 3065 (CH-Ar), 2971 & 2917 (CH-aliphatic), 2366, 1664 (C = O), 1601 (C = O), 1552 & 1493 (C = C), 1467, 1362, 1335, 1299, 1264, 1205, 1112, 1030, 962, 881, 768, 695, 641, 595, 568, 523, 468; δ_H_ (850 MHz; CDCl_3_) 8.66 (1H, br. s, NH), 8.27 (1H, d, *J*= 7.7, CH-Ar), 7.76 (1H, t, *J*= 7.7, CH-Ar), 7.62 (1H, d, *J*= 7.7, CH-Ar), 7.59–7.58 (2H, m, 2 × CH-Ar), 7.47 (1H, t, *J*= 7.7, CH-Ar), 7.33–7.32 (2H, m, 2 × CH-Ar), 7.11–7.07 (2H, m, 2 × CH-Ar), 7.04 (2H, d, *J*= 6.8, 2 × CH-Ar), 3.99 (2H, s, CH_2_), 2.18 (6H, s, 2 × CH_3_); δ_C_ (312 MHz; CDCl_3_) 166.94 (C = O), 161.33 (C = O), 157.78 (C = N), 146.99, 135.26, 135.23, 135.19, 133.55, 130.55, 130.05, 129.02, 128.27, 127.66, 127.38, 126.75, 125.73, 119.91 (12 × CH-Ar, 6 × C_q_-Ar), 35.49 (CH_2_), 18.33 (2 × CH_3_); MS (EI) *m/z* (%) [M^+^ + 1] 416.15 (1.05), [M^+^] 415.15 (2.86), 400.10 (9.45), 296.05 (20.13), 295.05 (100.00), 268.05 (28.58), 253.05 (10.77), 235.10 (30.01), 221.05 (5.97), 177.05 (7.25), 162.00 (2.47), 148.05 (3.93), 132.10 (20.59), 119.05 (11.21), 105.10 (2.87), 91.05 (12.43). Anal. calcd. for C_24_H_21_N_3_O_2_S (415.14): C, 69.37; H, 5.09; N, 10.11. Found: C, 69.18; H, 4.92; N, 10.00.

### N-(2,6-Dimethyl-phenyl)-2-(6-methyl-4-oxo-3-phenyl-3,4-dihydro-quinazolin-2-ylsulfanyl) -acetamide (15d)

White powder, yield (57%), mp 229–230 °C; ν_max_ (KBr)/cm^−1^ 3269 (NH), 3024 (CH-Ar), 2961 & 2919 (CH-aliphatic), 2372, 2341, 1688 (C = O), 1651 (C = O), 1574 & 1547 (C = C), 1486, 1408, 1336, 1296, 1260, 1199, 1103, 1032, 963, 912, 836, 774, 748, 692, 648, 604, 543, 504; δ_H_ (500 MHz; CDCl_3_) 8.70 (1H, br. s, NH), 8.05 (1H, s, CH-Ar), 7.57–7.49 (5H, m, 5 × CH-Ar), 7.32–7.30 (2H, m, 2 × CH-Ar), 7.07–7.02 (3H, m, 3 × CH-Ar), 3.96 (2H, s, CH_2_), 2.46 (3H, s, CH_3_), 2.16 (6H, s, 2 × CH_3_); δ_C_ (125 MHz; CDCl_3_) 166.99 (C = O), 161.39 (C = O), 156.66 (C = N), 145.07, 136.98, 136.57, 135.42, 135.18, 133.64, 130.46, 130.01, 129.08, 128.25, 127.32, 127.05, 125.59, 119.64 (11 × CH-Ar, 7 × C_q_-Ar), 35.49 (CH_2_), 21.30 (CH_3_), 18.35 (2 × CH_3_); MS (EI) *m/z* (%) [M^+^ + 1] 430.10 (1.24), [M^+^] 429.15 (3.40), 414.10 (9.75), 309.05 (100.00), 282.05 (35.11), 267.05 (10.35), 249.10 (38.79), 235.05 (5.16), 205.05 (1.86), 177.05 (5.60), 160.05 (2.87), 146.10 (16.74), 133.10 (10.36), 121.10 (4.97), 104.05 (10.15), 91.05 (11.20), 77.00 (21.32), 59.00 (3.71). Anal. calcd. for C_25_H_23_N_3_O_2_S (429.15): C, 69.91; H, 5.40; N, 9.78. Found: C, 69.76; H, 5.32; N, 9.64.

### N-(2,3-Dimethyl-phenyl)-2-(6-methyl-4-oxo-3-phenyl-3,4-dihydro-quinazolin-2-ylsulfanyl) -acetamide (15f)

White powder, yield (76%), mp 218–220 °C; ν_max_ (KBr/cm^−1^ 3259 (NH), 3000 (CH-Ar), 2925 (CH-aliphatic), 2371, 2341, 1692 (C = O), 1648 (C = O), 1574 & 1545 (C = C), 1487, 1300, 1263, 1204, 1147, 963, 834, 787, 750, 694, 651; δ_H_ (500 MHz; CDCl_3_) 8.90 (1H, s, NH), 8.06 (1H, s, CH-Ar), 7.57–7.53 (5H, m, 5 × CH-Ar), 7.49 (1H, d, *J*= 7.4, CH-Ar), 7.32–7.25 (2H, m, 2 × CH-Ar), 7.08 (1H, apparent t, *J*= 7.3, CH-Ar), 6.97 (1H, d, *J*= 6.9, CH-Ar), 3.97 (2H, s, CH_2_), 2.48 (3H, s, CH_3_), 2.23 (3H, s, CH_3_), 2.01 (3H, s, CH_3_); δ_C_ (125 MHz; CDCl_3_) 167.11 (C = O), 161.38 (C = O), 156.74 (C = N), 145.11, 137.41, 136.94, 136.50, 135.39, 135.31, 130.46, 129.96, 129.10, 128.95, 127.31, 127.05, 125.93, 125.67, 121.93, 119.66 (11 × CH-Ar & 7 × C_q_-Ar), 36.14 (CH_2_), 21.30 (CH_3_), 20.55 (CH_3_), 13.82 (CH_3_); MS (EI) *m/z* (%) [M^+^] 429.15 (0.15), 414.10 (0.65), 309.05 (100.00), 282.05 (5.52), 269.10 (6.32), 249.10 (13.83), 235.05 (3.23), 189.05 (1.52), 161.10 (1.76), 146.10 (10.05), 133.10 (6.23), 113.10 (7.77), 101.10 (4.81), 77.00 (13.43), 59.00 (21.59). Anal. calcd. for C_25_H_23_N_3_O_2_S (429.15): C, 69.91; H, 5.40; N, 9.78. Found: C, 69.85; H, 5.35; N, 9.75.

### Biological evaluation

#### Cell culture

Human CRC cell lines, HT-29 and SW620 cells were grown in RPMI (Invitrogen) containing 10% heat-inactivated foetal bovine serum, 100 µg/ml streptomycin, 100 units/ml penicillin and 2 mmol/l L-glutamine.

#### Cell viability assay

Cell viability was determined using the 3-(4, 5-dimethylthiazolyl-2)–2, 5-diphenyltetrazolium bromide) MTT assay according to Mosmann^40^. 5-Fluorouracil (5-FU) was used as positive control. Briefly, after culturing cells in 96 well plates for 24 h, they were treated with different compounds (30 μg/ml) for 24 h. Freshly prepared MTT (5 mM) solutions were added to the cells and were further incubated for 2 h at 37 °C in 5% CO_2_. One hundred microliters of dimethyl sulphoxide (DMSO) was added in each well to dissolve the formazan crystal, formed in the reaction during incubation by careful pipetting. The absorbance of the product was measured at 540 nm using a microplate reader. The experiments were performed in triplicate for each condition. The graph illustrates the mean and standard deviation (SD) values of three independent experiments.

#### Cytotoxicity assay using the xCELLigence system

The optimal seeding concentration for proliferation of HT-29 was determined. HT-29 cells (5000 cell in 150 µl medium/well) and SW620 cells (12,000 cell in 150 µl medium/well) were seeded in 16 well plates (E-plate 16-ACEA Biosciences Inc., San Diego, CA) as described in the xCELLigence Real Time Cell Analyzer (RTCA) DP instrument manual provided by the manufacturer. After 24 h, 20 and 40 µg/ml of compound **15d** were added and the experiments were allowed to run for another 3 d. Baseline cell indexes were calculated for at least two measurements from three replicate experiments.

#### Western blotting

Whole cell lysates were prepared using radioimmunoprecipitation assay (RIPA) lysis buffer as described by Al-Khayal and co-workers^41^. Total protein concentration was determined using Bradford protein reagent (Bio-Rad, Hercules, CA). Soluble proteins were loaded on precast TGX gels and were analysed using immunoblotting with anti-Bcl2, anti-BclxL (dilution 1:1000; Santa Cruz Biotechnology, Dallas, TX), and anti-β-actin (1:10,000 Sigma, St. Louis, MO). Reactivity was detected using horseradish peroxidase-conjugated secondary antibodies and chemiluminescence by Clarity Western ECL substrate (Bio-Rad). Membrane was developed using C-Digit Blot Scanner (LI-COR, Hamburg, Germany).

#### In vitro evaluation of antiphospholipases activities

The inhibitory activities of the synthesised *3H*-quinazolin-4-one derivatives against a group of phospholipases were investigated according to the method of De Aranjo and Radvany^42^ using five sPLA_2_s: human group IIA (hG- IIA), human group V (hG-V), human group X (hG-X), *Apis mellifera* bee venom (AmPLA_2_) and *Naja mossambica mossambica* sPLA_2_ (NmPLA_2_). Ten microliters of each sPLA_2_ solution (20 μg/ml) was mixed with 10 μl of each compound at different concentrations (0–50 μg/ml) and the mixture was incubated for 20 min at room temperature. Then, 1 ml of the PLA_2_ substrate (3.5 mM lecithin suspended in 100 mM NaCl, 10 mM CaCl_2_, 3 mM NaTDC, and 0.055 mM red phenol, pH 7.6) was added. The hydrolysis kinetics was followed spectrophotometrically for 5 min at 558 nm. The results were reported as the inhibition percentage that was calculated by comparison with a control experiment (absence of compound) and the IC_50_ values were determined from the curve. Oleanolic acid (Sigma) was used as the positive control.

#### In vitro evaluation of antiproteases activities

The inhibitory effects of 21 *3H*-quinazolin-4-one derivatives on several available therapeutically important proteases such as cathepsin-B, collagenase, elastase, thrombin, and trypsin were investigated. The inhibition of the enzymes in the presence of test compounds was evaluated by adding different concentrations of each compound (0–75 μg/ml) to the respective reaction mixture, preincubating for 15 min and then measuring the remaining enzyme activity as previously described^43^. The protease inhibitory activity was expressed as inhibition percentage, which was determined by comparison with a control experiment. The results were expressed as IC_50_ values that were determined from the standard curve. A protease inhibitor cocktail (Sigma) was used as the positive control.

#### In vitro inhibition of α-amylase activity

The α-amylase inhibitory activity of the tested compounds was investigated according to the reported method by Subranian^44^. Briefly, 10 µL of α-amylase (3,3 U) (Sigma) was mixed with 10 µl of different concentrations (20–200 µg/ml) of each compound, the appropriate solvent, or quercetin (positive control), and incubated at 37 °C for 5 min. After adding 180 µl of Labtest (amylase substrate), the samples were incubated for 8 min and the absorbance of first reaction was measured at 620 nm. Then, 100 µl of the reaction mixture was incubated for more five additional minutes at 37 °C and the absorbance of the second reaction was measured. Labtest was diluted in distilled water (1:1) before being added to the microplate. The concentration of quercetin used was same as those of the test compounds. The α-amylase inhibition percentage was calculated as follows: % inhibition = 100 _ (X2 sample _ X1 sample/X2 control _ X1 control) × 100 where X1 is the absorbance of the initial reading and X2 is the absorbance of the final reading. The results were expressed as IC_50_ determined from the curve.

#### In vitro inhibition of α-glucosidase activity

The α-glucosidase inhibitory activity of the tested compounds was evaluated according to the method described by Andrade-Cetto and collaborators^45^ based on the release of 4-nitrophenol α-D-glucopyranoside (4 NPGP). Briefly, 20 µl of different concentrations (ranging from 0 to 50 µg/ml) of each compound, the appropriate solvent, or quercetin (positive control) was mixed with 180 µl of the α-glucosidase enzyme from *Saccharomyces cerevisiae* (Sigma) and the obtained mixture was incubated at 37 °C for 2 min, followed by additional incubation for more than 15 min at 37 °C after the addition of 150 µl of the colour reagent NPGP. The colorimetric assay included 2 U of α-glucosidase, 5 mM of 4-NPGP, and 10 mM potassium phosphate buffer, pH 6.9. The concentration of quercetin used was identical to that of the tested compounds. The reading assay was performed using a microplate reader at 405 nm. The α-glucosidase inhibition percentage was calculated as follows: % inhibition = 100 _ (X2 sample _ X1 sample/X2 control _ X1 control) × 100 where X1 is the absorbance of the initial reading and X2 is the absorbance of the final reading, control is the absorbance of the assay with the appropriate solvent. The results were expressed as IC_50_ determined from the curve.

#### In vitro inhibition of xanthine oxidase

The xanthine oxidase inhibitory activities of the examined chemical compounds were determined by following the formation of uric acid from xanthine according to the reported method by Bondet and co-workers^46^. Forty microliters xanthine oxidase (667 mM) and 15 µl of each compound (0–150 µg/ml), allopurinol (positive control), or appropriate solvent (negative control) were added to each microplate well and preincubated for 5 min at 37 °C. Then, 95 µl of reagent 1 (mixture of hydroxylamine (0.2 mM), EDTA (0.1 mM), and xanthine oxidase (667 mM) in 50 mM phosphate buffer solution (pH 7.5) were added to the reaction mixture and incubated at the same temperature for 30 min. Subsequently, the absorbance was measured at 295 nm using a microplate reader. Finally, after addition of 150 µl of uric acid reagent, the absorbance was measured again. The concentration of allopurinol used was same as that of the tested compounds. Xanthine oxidase inhibition was calculated as follows: % inhibition = 100 _ (X2 sample _ X1 sample/X2 control _ X1 control) × 100 where X1 is the absorbance of the initial reading and X2 is the absorbance of the final reading. The results were expressed as IC_50_ determined from the curve.

## Results and discussion

### Chemistry

The main targets of this investigation were 3*H*-quinazolin-4-one derivatives possessing general structure **1**.

Two synthetic approaches were used for the synthesis of these scaffolds.

**Scheme 1.**
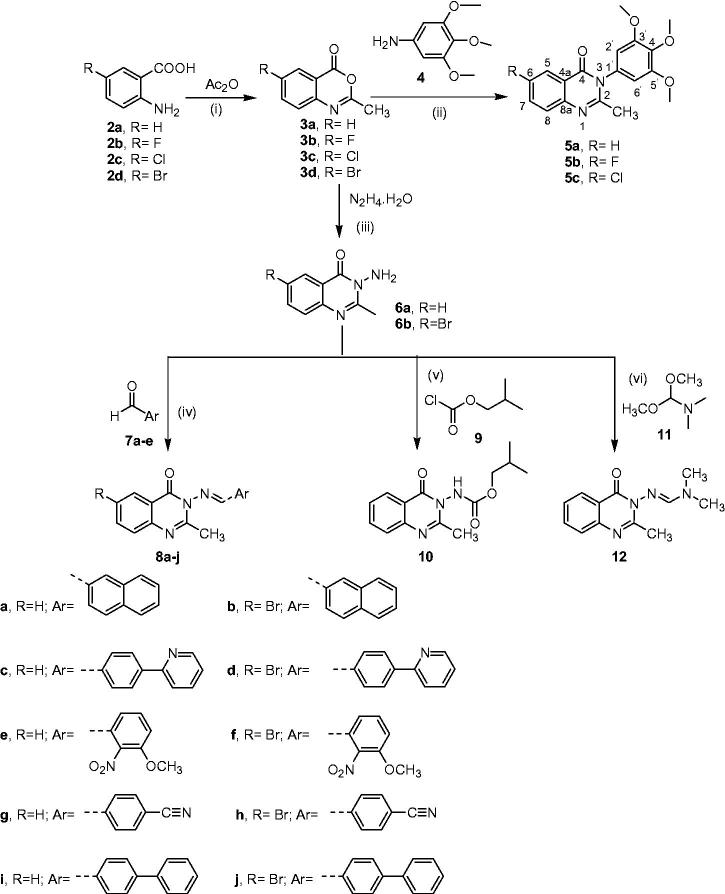
(i) Heating in domestic microwave at 540 watt for 8 min, cooling, and washing with petroleum ether; (ii) Fusion in oil bath at 200 °C, for 2 h, cooling to 100 °C, treatment with 37% HCl (Conc) till dissolution of the reaction mixture; cooling to room temperature and alkalisation with dilute NaOH (25%); (iii) ethanol, reflux for 8 h; (iv) ethanol and acetic acid catalytic, reflux for 10 h; (v) reflux for 10 h; (vi) ethanol, reflux for 2 h.

The first synthetic approach featured the preparation and chemical transformations of the key intermediates benzo[*d*][1,3]oxazin-4-one derivatives **3a–d** to various 3*H*-quinazolin-4-one derivatives as depicted in Scheme 1.

The conventional methods available for the synthesis of compounds **3a–d** *via* cyclocondensation of anthranilic acid derivatives **2a–d** with acetic anhydride^26–28^ were time consuming, therefore, these compounds were synthesised using the more efficient microwave-assisted method. Fusion of oxazinones **3a–c** with 3,4,5-trimetrhoxyanline **4** ^32^ ^,^ ^33^ yielded 2-methyl-3–(3,4,5-trimethoxy-phenyl)-3*H*-quinazolin-4-one derivatives **5a–c**. The assigned molecular structures of these new quinazolin-4-one derivatives were in full agreement with their spectroscopic and elemental analyses. Thus the IR spectrum (KBr) of compound **5c**, for example, displayed absorption bands at *v*_max_ 3075, 2934; 2839, 1680, 1598; 1504 and 834 cm^−1^ for aromatic-CH, aliphatic-CH, the carbonyl group, C = C and C–Cl, respectively. The ^1^H NMR spectrum of compound **5c** revealed the presence of five aromatic protons, which were displayed as; a one proton doublet signal at *δ* 8.18 ppm with coupling constant *J* of 2.4 Hz corresponding to CH^5^-quinazolin-4-one, a one-proton double-doublet signal at *δ* 7.69 ppm with coupling constant *J* of 8.7, 2.4 Hz due to CH^7^-quinazolin-4-one, a one-proton doublet signal at *δ* 7.60 ppm with coupling constant *J* of 8.7 Hz corresponding to CH^8^-quinazolin-4-one, and a two-protons singlet peak at *δ* 6.51 ppm, which was attributed to the two methine protons of 3,4,5-trimethoxyphenyl moiety (H2^'^ and H^6'^). The nine protons of the three methoxy groups were displayed as a three-protons singlet at *δ* 3.92, and a six-protons singlet at *δ* 3.88 ppm. Finally, the three protons of the methyl group were characterised by a three-protons singlet signal at *δ* 2.32 ppm. Moreover, the ^13^C NMR spectrum also confirmed the assigned structure based on the emergence of fifteen spectral lines at *δ_C_*161.28 (CO), 154.75 (C = N), 154.25, 145.91, 138.56, 135.02, 132.95, 132.36, 128.52, 126.23, 121.73, 105.09 (5 × CH-Ar & 7 × C_q_-Ar), 60.93 (OCH_3_), 56.28 (2 × OCH_3_) and 24.02 (CH_3_-quinazolin-4-one). The mass spectrum (EI) of compound **5c** indicated the presence of the expected molecular ion peak [M^+^] at *m/z* 360.10 *(*100%) for C_18_H_17_^35^ClN_2_O_4_.

Refluxing of oxazinones **3a**,**d** with excess hydrazine hydrate in ethanol yielded 3-aminoquinazolinons **6a**,**b** ^29^ ^,^ ^30^, which were converted to certain novel Schiff bases **8a–j** *via* condensation with a series of aryl aldehydes **7a–e** in absolute ethanol containing catalytic amount of glacial acetic acid^34^. The structures of the new Schiff’s bases were verified by their IR, ^1^H NMR, ^13^C NMR, mass spectroscopic (MS) data, and CHN analyses. The IR spectra of compounds **8a–j** indicated disappearance of the stretching bands of the amino groups in 3-aminoquinazolin-4-one derivatives **6a**,**b**. The ^1^H NMR spectra were characterised by the disappearance of the two-protons singlet signals corresponding to the amino protons of starting materials, which resonated at *δ* 5.79 and 4.89 ppm, accompanied by emergence of new one-proton singlet signals attributed to azomethine protons (CH = N) at *δ* 9.41^_^8.70 ppm range. In addition, the ^13^C NMR spectra displayed the expected spectral lines for each compound with the signals corresponding to (CH = N) groups resonating at *δ_C_* 159.10–156.46 ppm.

Furthermore, the amino group of 3-amino-2-methyl-3*H*-quinazolin-4-one **6a** was transformed to the corresponding carbamate group *via* direct nucleophilic substitution reaction for isobutyl chloroformate **9** ^35^ to yield the new carbamate derivative **10**. The structure of the latter product was established on the basis of its IR, ^1^H NMR, ^13^C NMR, MS and HCN elemental analyses. The IR spectrum displayed the characteristic stretching absorption bands at *v*_max_ 3223, 1755, and 1694 cm^−1^ indicating the presence of NH and two C = O groups. The ^1^H NMR spectrum of compound **10** was characterised by the presence of a one-proton broad singlet peak at *δ* 7.62 ppm due to NH group, in addition to the presence of a two-protons apparent singlet signal, a one-proton multiplet, and a six-protons doublet signal with coupling constant value *J* of 6.0 Hz at *δ* 4.00, 1.96–1.93 and 0.94 ppm, respectively corresponding to (O-CH_2_-CH-(CH_3_)_2_) isobutoxy group. Moreover, the ^13^C NMR spectrum of carbamate **10** revealed the emergence of a new spectral line at *δ_C_*156.28 ppm attributable to the C = O of the carbamate functional group. The methylene, methine and two methyl groups of isobutoxy moiety demonstrated three spectral lines at *δ_C_* 72.71, 27.78, and 18.72 ppm, respectively. Finally, the mass spectrum (EI) of compound **10** showed the anticipated molecular ion peaks [M^+^ + 1] at *m/z* 276.10 (21.00%) for C_14_H_17_N_3_O_3_ and [M^+^] 275.10 (100.00%).

Next, 3-amino-2-methyl-3*H*-quinazolin-4-one **6a** was transformed to the corresponding *N*,*N*-dimethylformamidine derivative **12** by refluxing with excess *N,N*-dimethyl formamide dimethyl acetal (DMF-DMA) **11** in absolute ethanol^36^. The structure of this new compound was deduced from its IR, ^1^HNMR, ^13^CNMR, MS data, and elemental analyses. The IR spectrum revealed the presence of absorption bands at *v*_max_ 3010, 2924; 2807, 1660, 1621, 1589; 1475 cm^−1^ which were attributed to CH-aromatic, CH-aliphatic, C = O, C = N, and C = C, respectively. In addition, the ^1^H NMR spectrum of formamidine **12** was characterised by the presence of a new one-proton singlet signal at *δ* 7.74 ppm attributed to azomethine proton (HC = N) and two singlet signals, each integrating to three protons resonating at *δ* 3.08 and 3.04 ppm, corresponding to the two methyl groups in dimethylamino N(CH_3_)_2_ moiety. Furthermore, the ^13^C NMR spectrum of compound **12** demonstrated three new peaks at *δ_C_* 160.08, 40.88, and 34.53 ppm which were attributed to HC=N-N(CH_3_)_2_ and, two carbons of the methyl groups in N(CH_3_)_2_ moiety, respectively. The mass spectrum (EI) of compound **12** showed the anticipated molecular ion peak [M^+^] at *m/z* 230.00 (34.52%) for C_12_H_14_N_4_O.

**Scheme 2.**
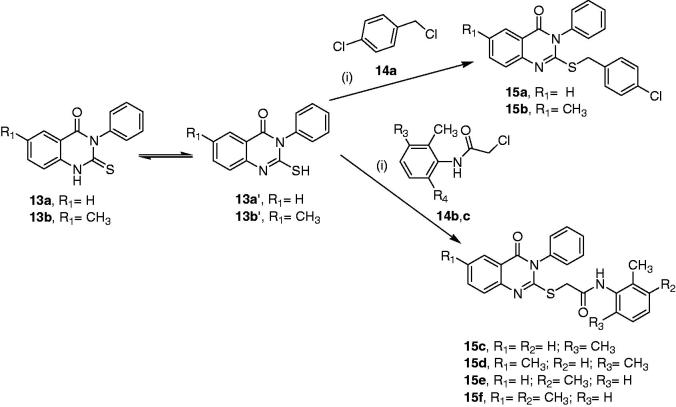
Reagents and conditions: (i) K_2_CO_3_ and dry acetone, reflux for 6–8 h.

The second synthetic approach for preparation of 3*H*-quinazolin-4-one derivatives involved the synthesis of compounds **15a–f** *via* nucleophilic attack by 2-mercapto-3-phenyl-3*H*-quinazolin-4-one derivatives **13a'**,**b'** on 4-chlorobenzyl chloride **14a** or chloroacetamide derivatives **14b**,**c** ^37^ ^,^ ^38^ to yield compounds **15a-f** by adapting modified procedure to what was reported by Salman et al.^39^ as illustrated in Scheme 2.

Structures of the new quinazolinone derivatives **15b** and **15d**,**f** were verified based on their respective IR, ^1^H NMR, ^13^C NMR, MS data, and elemental analyses. The IR spectra (KBr) of these products were characterised by the disappearance of the absorption bands at *v*_max_ 3245 and, 3169 cm^−1^ for the NH group (NH, SH exchangeable) and at *v*_max_ 1195 cm^−1^ for the thiocarbonyl group (C = S) of starting substrates **13a**,**b**. Furthermore, their ^1^H NMR spectra indicated the disappearance of the signals at *δ* 13.05 and 12.99 ppm due to the thiol protons of the starting materials. In addition, the spectrum of compound **15b** showed characteristic two-protons singlet signal at δ 4.32 corresponding to the benzylic methylene group. Analogously, the spectra of compounds **15d**,**f** were characterised by the emergence of new one-proton broad singlet signals at *δ* 8.70 and 8.90 ppm corresponding to NH groups, and two-protons singlet signals at *δ* 3.96 and, 3.97 belong corresponding to methylene groups. In addition, the two methyl groups of the acetamide moieties were represented by a six-protons singlet signal at *δ* 2.16 ppm for compound **15d**, and two singlet signals each integrating to three-protons at *δ* 2.23 and, 2.01 ppm for compound **15f**.

Furthermore, the ^13^C NMR spectra of compounds **15b**,**d**,**f** revealed the absence of spectral lines at *δ_C_* 176.05 and 176.00 ppm corresponding to the thiocarbonyl group in the starting materials **13a**,**b** and the presence of new peaks at *δ_C_* 36.22, 35.49, and 36.14 ppm attributed to the new methylene groups. The carbonyl and methyl groups of the acetamide moieties in compounds **15d**,**f** were observed at *δ_C_* 166.99, 167.11, 18.35, 20.55, and 13.82, respectively. In addition, the mass spectra (EI) of compounds **15b**,**d**,**f** displayed the anticipated molecular ion peaks.

### Biological evaluation

#### Effects of the synthesised 3H-quinazolin-4-one derivatives on cell viability of human colorectal cancer cell lines HT-29 and SW620

As CRC starts with initial benign stages advanced to late stage leading to metastasis. Therefore, two human CRC cell lines which belong to two different stages namely, HT-29 which is derived from adenocarcinoma CRC and SW620 which is derived from metastatic site were selected for *in vitro* evaluation of the cytotoxicity (30 μg/ml) of twenty-one compounds incorporating the 3*H*-quinazolin-4-one scaffold.

Cell viability was monitored using the MTT assay and the results are presented in Supplementary Figure 1. Compared to the negative control, and the positive control (5-FU), the synthesised 3*H*-quinazolin-4-one derivatives displayed varied degrees of cytotoxicity against the examined colorectal cell lines. Eight out of all the examined compounds showed significant decrease in cell viability. In one hand, compound **15c** inhibited only HT-29 cell line (47.5% viability) whereas compound **5b** inhibited only SW620 cell line (46.6% viability). On the other hand, six compounds namely, **8a**, **8f**, **8j**, **15a**, **15b**, and **15d**, were potently capable of inhibiting cell viability of both human CRC cell lines. Compound **15d** was the most active in this set reducing the cell viabilities of HT-29 and SW620 cells lines to 38% and, 36.7%, compared to 5-FU which demonstrated cell viabilities of 65.9 and 42.7%, respectively. Hence, compound **15d** was selected for further mechanistic studies. The results indicated that compound **15d** inhibited cell viability in a dose-dependent manner. The IC_50_ for **15d** was ∼20 µg/ml for HT-29 and SW620 cells (Supplementary Figures 2 and 3). These results were again confirmed using the xCELLigence RTCA system, which measures the cytotoxic effect of inhibitors in real time at different time points and **15d** was found to inhibit the cell proliferation in human CRC HT-29 and SW620 cell lines in dose- and time-dependent manner (Supplementary Figures 4 and 5).

#### Evaluation of anti-apoptotic protein expression by compound 15d

SW620 cells were treated with various concentrations of **15d** for 24 h. Subsequently, cell lysates were immunoblotted with the indicated antibodies in order to investigate the mechanism *via* which this compound inhibited cell viability of human CRC cells. Results shown in Supplementary Figure 6 indicated that **15d** inhibited the anti-apoptotic proteins, Bcl2 and BclxL. These findings demonstrate that compound **15d** inhibited the cell viability of human CRC cells by down regulating the expression of Bcl2 and BclxL and triggering the apoptotic pathway.

#### In vitro evaluation of antiphospholipase A_2_ (PLA_2_) and antiproteases activities of the synthesised 3H-quinazolin-4-one derivatives

As inflammation is associated with several types of cancers *via* influencing growth, apoptosis, and proliferation of cancer and stromal cells, the synthesised 3*H*-quinazolin-4-one derivatives were further screened to evaluate their antiphospholipases activities against proinflammatory enzymes implicated in cancer namely, hGIIA, hGV, and X^10^. In addition, the inhibitory activity of these compounds towards the allergen and inflammatory PLA_2_ from honey bee venom (*Apis mellifera*, AmPLA_2_)^47^ and proinflammatory, heamolytic, myotoxic and neurotoxic Cobra (*Naja mosambique mosambique*) venom PLA_2_ (NmPLA_2_)^48^ were evaluated by determining their IC_50_ values. Results (Supplementary Table 1) revealed that six compounds, namely, **5a**, **8c**, **8d**, **8j**, **15a,** and **15d** displayed the most promising potency in inhibiting the catalytic activity of the studied hG-IIA, with IC_50_ values ranging from 5.75 ± 1.06 to 8.60 ± 0.56 µg/ml compared to that recorded with oleanolic acid which was used as a standard drug (5.25 ± 0.35 µg/ml). Among these compounds, **8d** exhibited the lowest IC_50_ value (5.75 ± 1.06 µg/ml).

The anti-PLA_2_ hGV data showed that three compounds; **5a**, **8d**, and **8j** inhibited the enzyme with IC_50_ values ranging from 6.25 ± 0.78 to 9.60 ± 0.85 µg/ml compared to the value recorded with the standard drug (oleanolic acid, IC_50_; 7.50 ± 0.71 µg/ml). Amongst them, compound **8j** (IC_50_; 6.25 ± 0.78 µg/ml), was being more potent than the reference drug.

The analysis of the inhibitory potency of the examined compounds against PLA_2_ hG-X showed that compounds; **5a**, **5 b**, **5c**, **8d**, **8j**, **10**, **15a,** and **15e** inhibited the enzymatic activity with IC_50_ values ranging from 6.60 ± 0.85 to 9.50 ± 0.71 µg/ml. It worth to note that compounds **8d**, **15a,** and **15e** exhibited lowered IC_50_ values than the reference drug (IC_50_; 7.55 ± 0.64 µg/ml).

Amongst the tested 3*H*-quinazolin-4-one derivatives which exhibited inhibitory activity against NmPLA_2_ were compounds; **5a**, **5b**, **5c**, **8c**, **8d**, **8e**, **8f**, **15a,** and **15d** with IC_50_ values ranging from 4.15 ± 0.50 to 8.50 ± 2.12 µg/ml compared to oleanolic acid (IC_50_; 4.90 ± 0.42 µg/ml). Interestingly, compound **5b** demonstrated the lowest IC_50_ which is also lower than that of the reference drug fusspot.

Finally, 13 compounds namely, **5a**, **5b**, **5c**, **8b**, **8c**, **8d**, **8e**, **8f**, **8i**, **10**, **15a**, **15d,** and **15e** effectively suppressed AmPLA_2_ with IC_50_ values ranging from 4.20 ± 0.57 to 9.40 ± 0.85 µg/ml compared to the IC_50 _ 4.20 ± 0.42 µg/ml value of oleanolic acid. Based on these results, AmPLA_2_ appears to be the maximally affected phospholipase by the synthesised compounds with compound **8f** (IC_50_; 4.20 ± 0.57 µg/ml) demonstrating inhibitory activity equivalent to that of the reference drug.

Similarly, the inhibitory potency of the selected 3*H*-quinazolin-4-one derivatives against five proteases namely, cathepsin-B, collagenase, thrombin, elastase, and trypsin was quantified by measuring and comparing the IC_50_ values produced by these compounds relative to the value produced by the protease inhibitor cocktail. The results of anti-proteases shown in Supplementary Table 2 indicated that compounds **8j** and **15e** were being the most active inhibitors against protease cathepsin-B with IC_50_ values of 1.40 ± 0.06 and 1.60 ± 0.07 µg/ml respectively compared to cocktail (IC_50_; 0.175 ± 0.04 µg/ml).

Furthermore, compounds **8b**, **8d,** and **15e** were found to be the most active in suppressing the collagenase activity with IC_50_ values ranging from 3.00 ± 0.01 to 4.50 ± 0.71 µg/ml compared to cocktail (IC_50_; 0.15 ± 0.07 µg/ml) with compound **8d** exhibiting the lowest IC_50_ value.

The highest anti-thrombin activity was demonstrated by compounds **5b**, **5c**, **8e**, **10**, and **15b**, with IC_50_ values ranging from 0.90 ± 0.14 to 2.50±0.71 µg/ml, with **5b** demonstrating the lowest IC_50_ compared to cocktail (IC_50_; 0.25 ± 0.07 µg/ml).

The highest anti-elastase activity was demonstrated by compounds **5b**, **5c**, **8e**, **10**, and **15b**, with IC_50_ values ranging from 0.65 ± 0.02 µg/ml (compound **15b**) to 1.75 ± 0.05 µg/ml (compound **5c**) compared to that of cocktail (IC_50_; 0.125 ± 0.04 µg/ml).

Finally, the lowest IC_50_ values ranging from 0.50 ± 0.00 to 2.25 ± 0.04 µg/ml against trypsin were demonstrated by compounds **5b**, **5c**, **8e**, **10**, and **15 b**, with compound **15b** displaying the lowest IC_50_ compared to that of the reference drug (IC_50_; 0.215 ± 0.04 µg/ml).

#### In vitro evaluation of inhibitory activities of 3H-quinazolin-4-one derivatives against enzymes implicated in metabolic syndrome

Considering an interactive relationship between diabetes and cancers, the *in vitro* inhibitory potential of the prepared 3*H*-quinazolin-4-one derivative against α-amylase, α-glucosidase, and xanthine oxidase (enzymes related to metabolic syndrome) was investigated.

The IC_50_ values exhibited by the chemical compounds against α-amylase activity are shown in Supplementary Table 3, which indicated that compounds **8d**, **8h**, **8j**, **15a**, **15b**, **15e**, and **15f**, were more potent in suppressing the enzyme with IC_50_ values ranging from 94.00 ± 2.83 µg/ml (compound **15b)** to 121.00 ± 9.90 µg/ml (compound **15e)** than quercetin the reference drug (IC_50_ 123.0 ± 2.83 µg/ml).

In addition, compounds **8g** (IC_50_ 3.25 ± 0.35 µg/ml), **8h** (IC_50_ 3.05 ± 0.21 µg/ml), and **8i** (IC_50_ 3.45 ± 0.64 µg/ml) were more potent in inhibiting α-glucosidase activity than quercetin (IC_50_ 3.80 ± 0.28 µg/ml) (Supplementary Table 4).

Although none of the tested compounds was more effective than allopurinol (reference drug) in inhibiting xanthine oxidase (XO) activity (Supplementary Table 5) (IC_50_ 0.65 ± 0.07 µg/ml), compound **8f** (IC_50_ 3.00 ± 1.41 µg/ml) was the most active among the tested compounds.

## Conclusions

A series of 25 compounds incorporating the 3*H*-quinazolin-4-one scaffold were synthesised , 20 of which were new and fully characterised. Twenty-one compounds were screened for their cytotoxicity on human CRC cell lines HT-29 and SW620 using the MTT assay. Six compounds namely, **8a**, **8f**, **8j**, **15a**, **15b**, and **15d** potently reduced viability of both cell lines, with **15d** the most active in this set displaying residual viability 38%, 36.7% against HT-29 and SW620 cells lines, respectively. Further mechanistic studies using **15d** revealed that this compound inhibited the cell proliferation of HT-29 and SW620 cell lines in a dose- and time-dependent manner with IC_50_ values ∼20 µg/ml. Evaluation of anti-apoptotic protein expression by compound **15d** revealed that it reduced the viability of the used human CRC cells by down regulating the expression of Bcl2 and BclxL.

Based on these results, **15d** could be considered as an attractive candidate for further investigations as a potential anticancer agent. Furthermore, the prepared compounds were screened for their inhibitory activities against certain phospholipases including, hG-IIA, hG-V, hG-X, NmPLA_2_, and AmPLA_2_, and five proteases namely, cathepsin-B, collagenase, thrombin, elastase, and trypsin, in addition to three enzymes involved in metabolic syndrome namely, α-amylase, α-glucosidase, and xanthine oxidase. Although none of the tested compounds showed good antiprotease activity, six compounds **8j**, **8d**/**15a**/**15e**, **5b**, and **8f** demonstrated higher antiphospholipases potential against proinflammatory isoforms hGV, hG-X, NmPLA_2_ and AmPLA_2_ than oleanolic acid, which was used as reference drug.

Therefore, these compounds would be of interest as preclinical candidates in trials for anticancer and anti-inflammatory drugs.

All the tested compounds were inactive against xanthine oxidase, whereas compounds **8d**, **8h**, **8j**, **15a**, **15b**, **15e**, and **15f** elicited higher α-amylase inhibition than the reference drug quercetin. Furthermore, compounds **8g**, **8h**, and **8i** were more potent in inhibiting α-glucosidase activity than the reference drug allopurinol. Consequently, these compounds can be considered as potential targets that can be further investigated for developing novel anti-diabetes mellitus (DM) drugs.

## Supplementary Material

Supplemental Material
