# Supplementary material for: Synthesis and evaluation of anticancer, antiphospholipases, antiproteases, and antimetabolic syndrome activities of some 3H-quinazolin-4-one derivatives
Source: J Enzyme Inhib Med Chem. 2019 Mar 1;34(1):672–83. doi: 10.1080/14756366.2019.1574780 (PMC6407576; doi:10.1080/14756366.2019.1574780)
Supplement: Supplemental Material [file IENZ_A_1574780_SM6806.docx]

**Synthesis and evaluation of anticancer, antiphospholipases, antiproteases, and antimetabolic syndrome activities of some 3*H-*quinazolin-4-one derivatives**

Nahed N. E. El-Sayed, Norah M. Almaneai, Abir Ben Bacha, Omar Al-Obeed, Rehan Ahmad, Maha Abdulla, and Ahmed M. Alafeefy

*Correspondence details: Nahed N. E. El-Sayed, Department of Chemistry, College of Science, King Saud University, P.O. Box 22452, Riyadh 11451, Saudi Arabia, nelsayed@ksu.edu.sa*

**
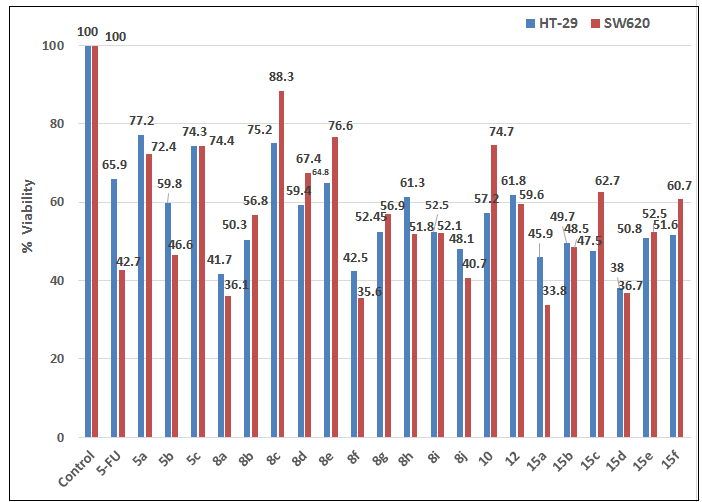
**

**Figure 1. The effects the synthesised 3*H*-quinazolin-4-one** **derivatives (30 μg/ml) on colorectal cancer HT-29 and SW620 cell lines**


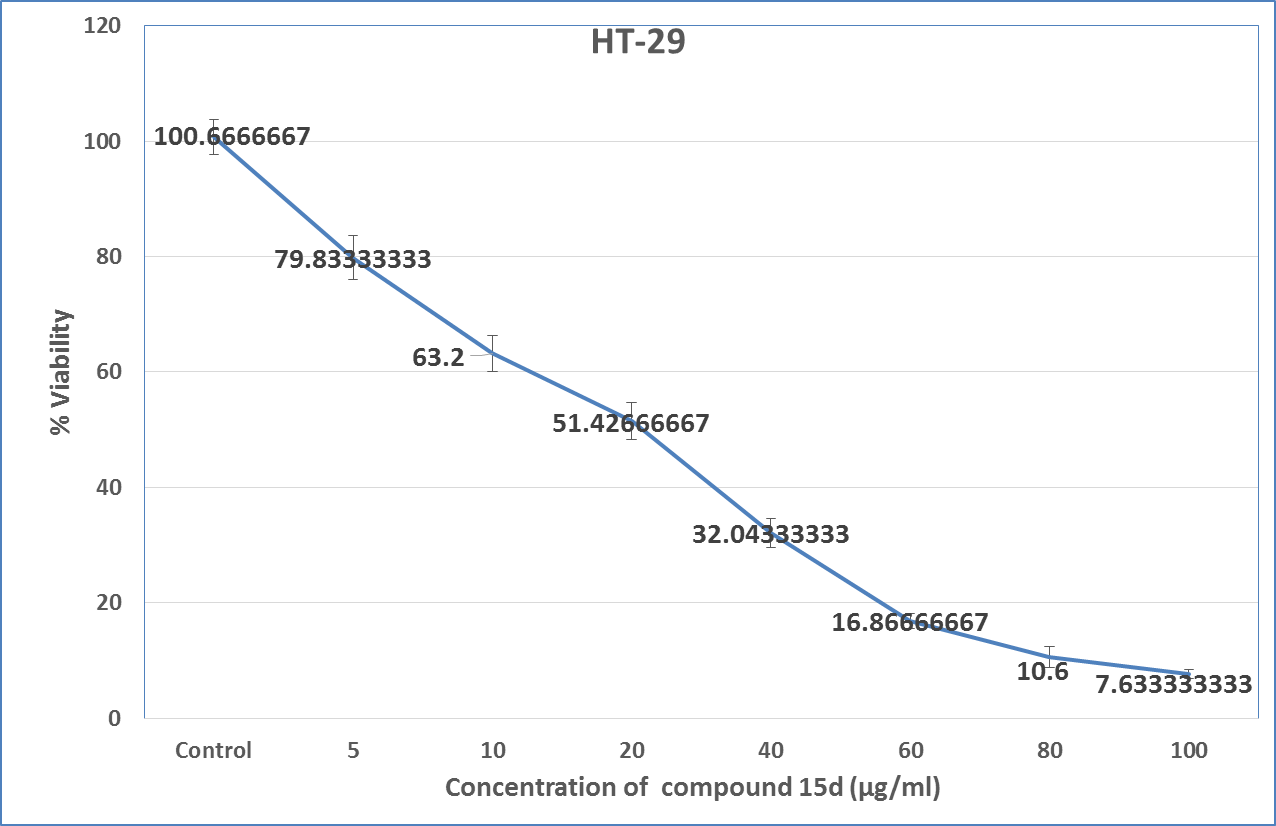


**Figure 2. Determination of the IC_50_ value for compound 15d on colorectal cancer HT‑29 cell line**

**
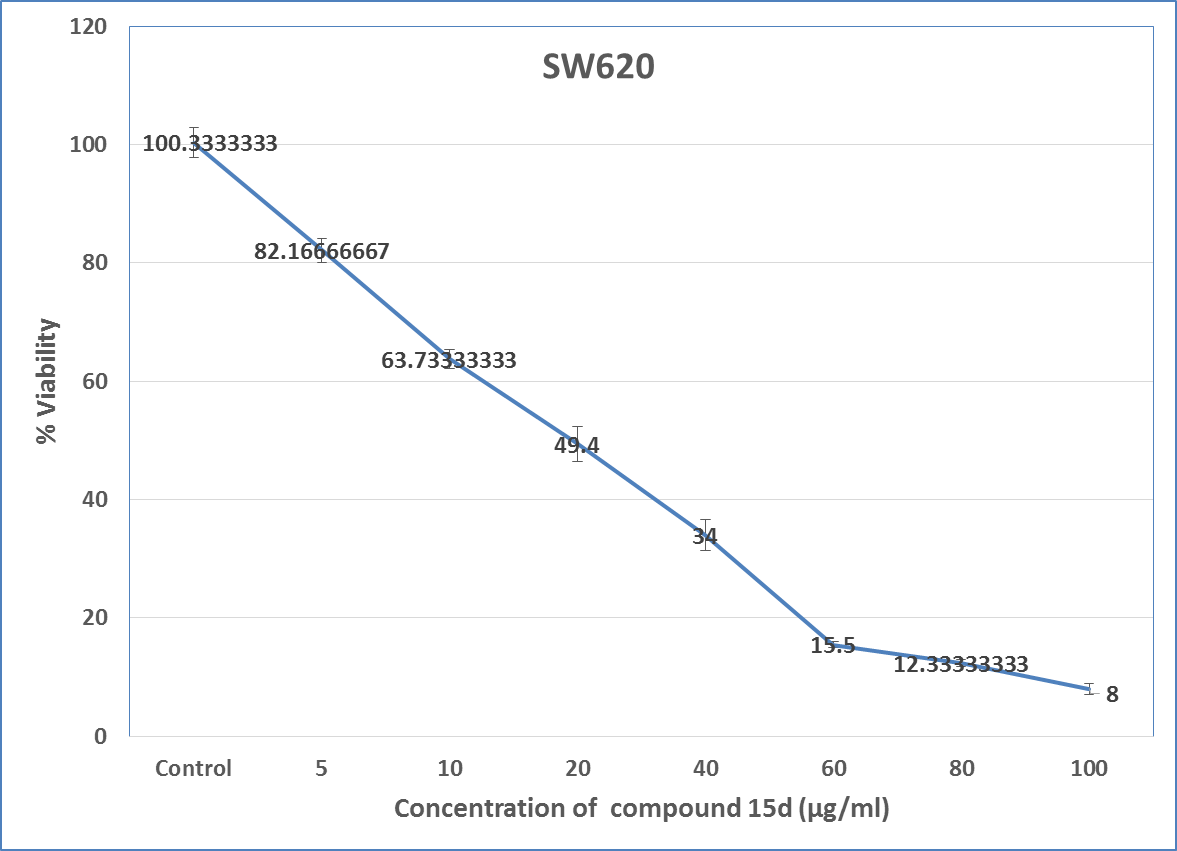
**

**Figure 3. Determination of the IC_50_ value for compound 15d on colorectal cancer SW620 cells**

**
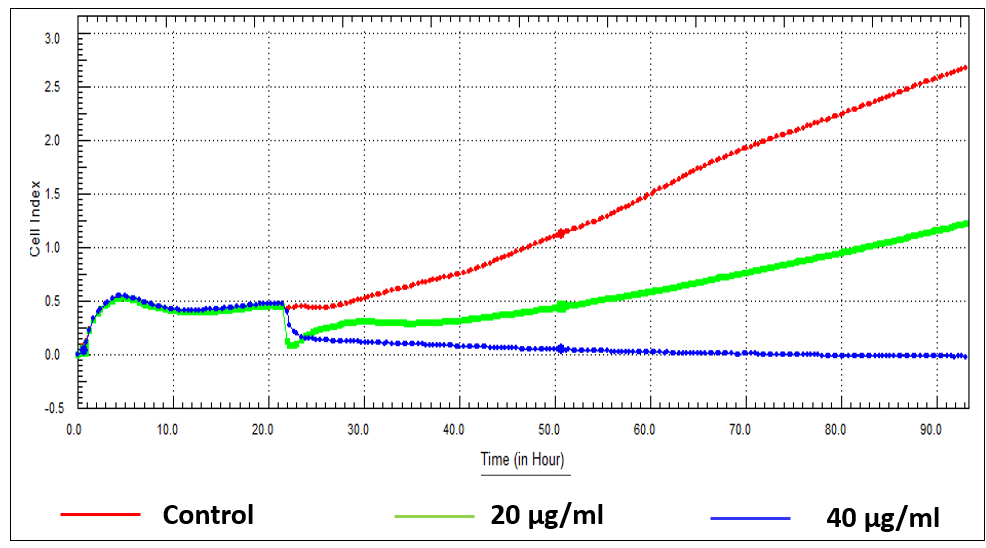
**

**Figure 4. The effects of different concentrations of compound 15d on colorectal cancer HT-29 cell line with time**

**
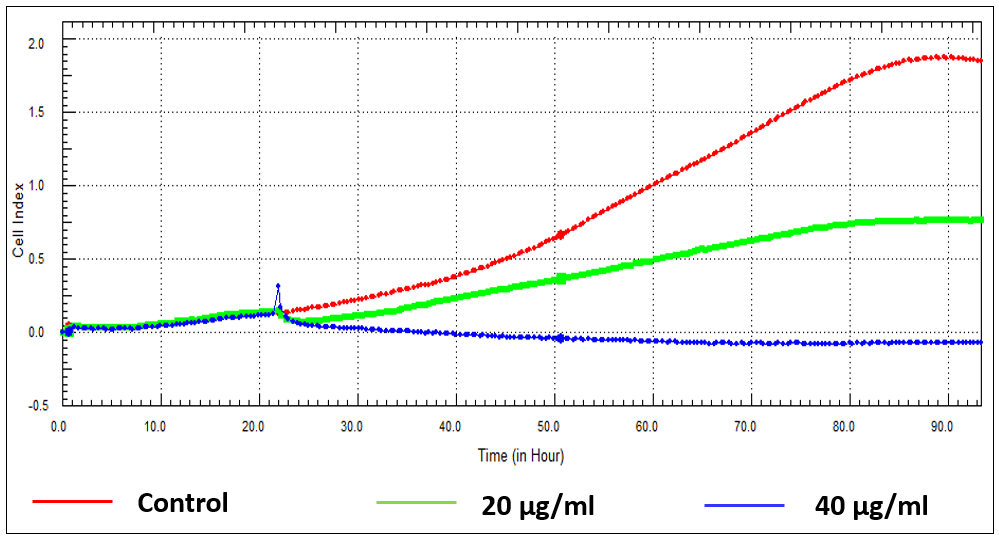
**

**Figure 5. The effects of different concentrations of compound 15d on colorectal cancer SW620 cell line with time**


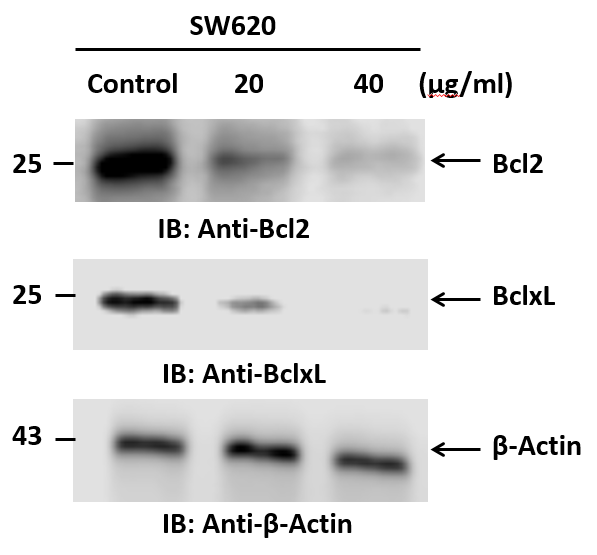


**Figure 6. Inhibition of anti-apoptotic proteins Bcl2 and BclxL by compound 15d**

| **Compound** | **hG-IIA** | **hG-V** | **hG-X** | **(NmPLA_2_)** | **AmPLA_2_** |
| --- | --- | --- | --- | --- | --- |
| **5a** | 7.00±1.41 | 9.60±0.85 | 7.65±0.92 | 6.00±0.71 | 5.50±0.71 |
| **5b** | 20.75±1.77 | 12.50±0.71 | 8.50±0.71 | 4.15±0.50 | 5.10±0.85 |
| **5c** | 20.75±0.35 | 13.75±1.06 | 9.50±0.71 | 5.15±0.92 | 4.55±0.64 |
| **8a** | 45.50±2.12 | 38.00±1.41 | 28.50±0.71 | 36.75±2.48 | 31.00±1.41 |
| **8b** | 16.60±0.85 | 21.50±2.12 | 13.00±1.41 | 16.25±1.06 | 7.25±0.35 |
| **8c** | 7.55±0.64 | 10.50±0.71 | 23.75±1.77 | 7.60±0.85 | 5.60±0.85 |
| **8d** | 5.75±1.06 | 9.35±0.92 | 6.60±0.85 | 6.55±0.78 | 4.40±0.57 |
| **8e** | 16.60±0.57 | 23.75±1.77 | 11.75±1.06 | 7.75±0.35 | 7.60±0.57 |
| **8f** | 18.60±0.57 | 15.75±1.06 | 18.00±1.41 | 6.00±0.71 | 4.20±0.57 |
| **8g** | 27.50±0.71 | 33.8±1.70 | 23.00±1.41 | 12.75±1.06 | 13.50±0.71 |
| **8h** | 44.00±1.41 | 34.00±1.41 | 41.00±1.41 | 22.90±1.27 | 29.00±1.41 |
| **8i** | 15.40±0.85 | 23.00±1.41 | 17.00±1.41 | 11.00±1.41 | 9.40±0.85 |
| **8j** | 8.45±0.78 | 6.25±0.78 | 9.45±0.78 | 16.40±0.85 | 19.00±1.41 |
| **10** | 15.50±0.71 | 17.25±1.06 | 9.50±0.71 | 13.45±0.78 | 4.50±0.71 |
| **12** | 46.00±1.41 | 34.50±0.71 | 32.50±2.12 | 35.00±2.83 | 28.00±2.83 |
| **15a** | 8.60±0.56 | 10.5±0.71 | 6.75±1.06 | 5.40±0.57 | 6.50±0.71 |
| **15b** | 26.00±1.41 | 18.40±0.85 | 13.50±0.71 | 14.85±0.50 | 13.60±0.85 |
| **15c** | 40.00±2.83 | 23.50±2.12 | 42.50±2.12 | 28.50±2.12 | 22.00±1.41 |
| **15d** | 7.15±1.20 | 10.50±0.71 | 23.50±2.12 | 8.50±2.12 | 6.00±1.41 |
| **15e** | 14.50±0.70 | 13.40±0.85 | 7.25±1.06 | 11.00±1.41 | 8.75±1.06 |
| **15f** | 39.00±1.41 | 30.10±1.56 | 22.00±2.82 | 33.00±2.83 | 37.25±1.06 |
| **Oleanolic acid** | 5.25±0.35 | 7.50±0.71 | 7.55±0.64 | 4.90±0.42 | 4.20±0.42 |

**Table 1. The mean IC_50_ values (µg/ml) of the synthesised 3*H*‑quinazolin-4-one** **derivatives against certain phospholipases**

| **Compound** | **Cathepsin-B** | **Collagenase** | **Thrombin** | **Elastase** | **Trypsin** |
| --- | --- | --- | --- | --- | --- |
| **5a** | 8.25±0.61 | 12.25±1.77 | 22.00±1.41 | 23.5±0.71 | 29.00±1.41 |
| **5b** | 11.00±1.41 | 18.00±1.41 | 0.90±0.14 | 0.70±0.01 | 1.10±0.14 |
| **5c** | 12.50±2.12 | 21.50±2.12 | 2.50±0.71 | 1.75±0.05 | 2.25±0.04 |
| **8a** | 36.50±2.12 | 31.00±1.41 | 34.00±1.41 | 32.50±2.12 | 34.50±0.71 |
| **8b** | 21.50±2.12 | 4.25±1.06 | 16.50±2.12 | 21.50±2.12 | 25.50±0.71 |
| **8c** | 12.50±2.12 | 23.50±0.71 | 23.50±0.71 | 32.00±2.83 | 35.00±1.41 |
| **8d** | 34.50±3.54 | 3.00±0.01 | 15.50±2.12 | 26.00±1.41 | 23.00±2.83 |
| **8e** | 2.35±0.50 | 15.50±2.12 | 1.50±0.07 | 1.25±0.05 | 0.85±0.02 |
| **8f** | 19.00±2.83 | 15.50±2.12 | 25.00±1.41 | 30.50±2.12 | 31.00±1.41 |
| **8g** | 7.25±1.77 | 17.25±1.77 | 14.00±1.41 | 11.00±0.41 | 13.50±0.71 |
| **8h** | 4.00±1.41 | 14.50±2.12 | 24.50±2.12 | 27.50±2.12 | 33.50±2.12 |
| **8i** | 3.50±0.71 | 15.25±1.06 | 17.50±2.12 | 13.50±2.12 | 12.50±2.12 |
| **8j** | 1.40±0.06 | 14.00±1.41 | 12.50±1.12 | 9.00±0.41 | 9.50±0.51 |
| **10** | 22.50±2.12 | 28.00±1.41 | 2.35±0.30 | 1.45±0.21 | 1.10±0.14 |
| **12** | 27.50±2.12 | 35.50±2.12 | 5.50±0.12 | 8.50±2.12 | 13.00±0.41 |
| **15a** | 18.50±2.12 | 28.00±1.41 | 13.50±2.12 | 11.50±2.12 | 12.00±1.41 |
| **15b** | 13.50±2.12 | 26.50±2.12 | 1.00±0.28 | 0.65±0.02 | 0.50±0.00 |
| **15c** | 11.00±1.41 | 18.00±1.41 | 17.50±2.12 | 18.50±0.71 | 15.50±0.71 |
| **15d** | 4.00±0.41 | 10.50±0.71 | 17.00±1.41 | 15.50±2.12 | 14.00±1.41 |
| **15e** | 1.60±0.07 | 4.50±0.71 | 24.00±1.41 | 31.50±2.12 | 34.00±1.41 |
| **15f** | 4.00±0.41 | 10.50±2.12 | 17.50±2.12 | 16.00±1.41 | 17.00±2.83 |
| **Protease Inhibitor Cocktail** | 0.175±0.04 | 0.15±0.07 | 0.25±0.07 | 0.125±0.04 | 0.215±0.04 |

**Table 2. The mean IC_50_ values of the synthesised 3*H*‑quinazolin-4-one** **derivative against some proteases**

| **Compound** | **Mean**  **IC_50_ against α‑amylase** |
| --- | --- |
| **5a** | 151.00 ±4.24 |
| **5b** | 131.00±5.66 |
| **5c** | 159.00±2.83 |
| **8a** | 165.50±6.36 |
| **8b** | 146.50±4.95 |
| **8c** | 142.50±7.78 |
| **8d** | 117.00±9.90 |
| **8e** | 143.50±7.78 |
| **8f** | 210.00±7.07 |
| **8g** | 182.00±4.24 |
| **8h** | 97.00±2.83 |
| **8i** | 199.50±6.36 |
| **8j** | 118.00±5.66 |
| **10** | 159.50±7.78 |
| **12** | 192.50±10.61 |
| **15a** | 121.00±8.49 |
| **15b** | 94.00±2.83 |
| **15c** | 184.00±7.07 |
| **15d** | 199.50±3.54 |
| **15e** | 121.00±9.90 |
| **15f** | 99.50±7.78 |
| **Quercetin** | 123.00±2.83 |

**Table 3. The mean IC_50_ values of the synthesised 3*H*‑quinazolin-4-one** **against α‑amylase**

| **Compound** | **Mean** |
| --- | --- |
| **5a** | 21.00±1.41 |
| **5c** | 24.50±0.71 |
| **5b** | 23.00±2.83 |
| **8a** | 16.40±1.98 |
| **8b** | 9.50±2.12 |
| **8c** | 5.00±1.41 |
| **8d** | 21.00±1.41 |
| **8e** | 5.60±0.57 |
| **8f** | 6.00±1.41 |
| **8g** | 3.25±0.35 |
| **8h** | 3.05±0.21 |
| **8i** | 3.45±0.64 |
| **8j** | 5.85±1.20 |
| **10** | 9.50±2.12 |
| **12** | 13.00±1.41 |
| **15a** | 5.00±1.41 |
| **15b** | 4.50±0.71 |
| **15c** | 30.40±1.98 |
| **15d** | 6.00±1.41 |
| **15e** | 7.00±1.41 |
| **15f** | 5.75±1.06 |
| **Quercetin** | 3.80±0.28 |

**Table 4. The mean IC_50_ values of the synthesised 3*H*‑quinazolin-4-one** **against α‑glucosidase**

| **Compound** | **Mean IC_50_** |
| --- | --- |
| **5a** | 7.25±1.06 |
| **5c** | 13.00±2.83 |
| **5b** | 5.40±1.98 |
| **8a** | 43.00±5.66 |
| **8b** | 10.00±2.83 |
| **8c** | 4.00±1.41 |
| **8d** | 10.00±1.41 |
| **8e** | 34.50±4.95 |
| **8f** | 3.00±1.41 |
| **8g** | 74.00±5.66 |
| **8h** | 56.50±3.54 |
| **8i** | 8.50±2.12 |
| **8j** | 20.50±3.54 |
| **10** | 30.00±4.24 |
| **12** | 21.00±2.83 |
| **15a** | 12.00±2.28 |
| **15b** | 19.50±2.12 |
| **15c** | 61.50±4.95 |
| **15d** | 71.00±4.24 |
| **15e** | 41.00±5.66 |
| **15f** | 58.50±4.95 |
| **Allopurinol** | 0.65±0.07 |

**Table 5. The mean IC_50_ values of the synthesised 3*H*‑quinazolin-4-one** **against xanthine oxidase**
